# Changes in Palatability Processing across the Estrous Cycle Are Modulated by Hypothalamic Estradiol Signaling

**DOI:** 10.1523/ENEURO.0225-25.2026

**Published:** 2026-03-10

**Authors:** Jian-You Lin, Bradly T. Stone, Oran M. Rahamim, Ainsley E. Craddock, Donald B. Katz

**Affiliations:** ^1^Neuroscience Program, Brandeis University, Waltham, Massachusetts 02453; ^2^Department of Psychology, Brandeis University, Waltham, Massachusetts 02453; ^3^Volen National Center for Complex Systems, Brandeis University, Waltham, Massachusetts 02453

**Keywords:** estradiol, estrous cycle, lateral hypothalamus, rat, taste palatability

## Abstract

Consumption varies across the stages (metestrus, diestrus, proestrus, estrus) of a rat's estrous cycle, changing in ways that might be expected to reflect, in part, a direct impact of hormones on taste palatability. Evidence regarding this hypothesis has been mixed, however, and critical within-subject experiments comparing consumption of multiple tastes with distinct valences across all estrous phases have been few. Here, we assayed female rats' licking of palatable (saccharin, sucrose, NaCl) and aversive (quinine-HCl, citric acid) tastes in brief-access trials, while tracking their estrous cycles through vaginal cytology. We observed sucrose palatability to be high at metestrus, the same phase at which the palatability of the aversive citric acid was low. These patterns were consistent across tastes of similar palatability, despite vast differences between the substances' receptor mechanisms and central impacts. Together, these results reveal a general (i.e., independent of particular tastant identity) magnification of palatability—higher than average for palatable tastes and lower for aversive tastes—centered largely on metestrus. We tested whether this phenomenon reflects lateral hypothalamic (LH) estradiol processing, by infusing LH with an estrogen receptor blocker (ICI182, 780) across five consecutive tasting sessions. Control infusions replicated the metestrus magnification of palatability; as predicted, ICI infusions blocked this effect, but estrogen receptor inhibition failed to render preferences “cycle free,” instead delaying the palatability magnification until diestrus. In summary, the estrous cycle directly mediates taste palatability in a manner involving hypothalamic actions of estradiol, but this effect is only one of several impacting consumption across the estrous cycle.

## Significance Statement

Consummatory behaviors are altered by numerous factors including naturally circulating hormones. While decades of work has investigated the role of reproductive hormones on consumption, it remains unclear whether (and to what degree) hormone-driven consumption variability is related to changes in central palatability processing. Here we show that taste palatability is indeed directly modulated by estrous phase—during metestrus, the differences between licking to palatable and aversive tastes is magnified—and go on to show that this phenomenon is governed, at least in part, by estradiol processing within the lateral hypothalamus.

## Introduction

Hormones influence animals' behavior in response to internal and external stimuli, notably impacting the amount of food/drink that animals consume (Martini et al., 1994; Eckel, 2004; Asarian and Geary, 2013; Duval et al., 2014; Rivera and Stincic, 2018). Humans, for instance, consume less nutrient-rich food during the midfollicular phase of their menstrual cycles and consume more during the midluteal phase (Tarasuk and Beaton, 1991; Martini et al., 1994). Similarly, a rat's consumption decreases ∼24 h following estradiol peaks (estrus), rising again 1–2 d later (i.e., metestrus/diestrus; Ter Haar, 1972; Fantino and Brinnel, 1986). In general, the estradiol peak in the human menstrual (and rat estrous) cycle appears to have a complex, delayed impact on consumption (Eckel, 2004; Asarian and Geary, 2013).

It is reasonable to hypothesize that such hormone-induced consumption fluctuations might, at least in part, reflect direct shifts in palatability (central decisions regarding the preferability or aversiveness of tasted substances). Support for this theory has been less than conclusive, however: some researchers suggest, on the basis of licking or orofacial data, that estradiol’s impact on sucrose and NaCl consumption is indeed palatability related (Curtis et al., 2004; Atchley et al., 2005; Rivera et al., 2007; Pereira Jr et al., 2019); others, however, argue that postingestive (i.e., post-taste) mechanisms are the cause of across-cycle consumption changes (Geary et al., 1995; Eckel, 2004).

Within-rat experiments comparing consumption of multiple tastes could potentially clarify this issue, because palatability is an intrinsically relational construct; the hypothesis that a change in consumption of a substance reflects a general change in palatability processing is difficult to test in the absence of comparisons of tastes with similar and distinct palatabilities. A few studies have performed such comparisons: Clarke and Ossenkopp (1998) examined taste reactivity and found only mixed evidence for palatability processing differences between high and low estradiol halves of the cycle; Parker et al. (2002), meanwhile, reported mild increases in consumption of sugary cereal during the period including metestrus and diestrus (see also Butcher et al., 1974; Cora et al., 2015), in the absence of comparable increases in consumption of less preferred chow. Clearly, and as reviews (Rivera and Stincic, 2018) explicitly note, more work of this sort is needed to resolve this issue.

Here, we analyze licking to a battery of tastes during each of the four estrous phases. By using the Brief Access Test (BAT; Boughter et al., 2002; St John and Spector, 2020; Schiff et al., 2023), we were able to compare licking to multiple distinct palatable and aversive tastes within single test sessions and thereby to estimate palatabilities (using average lick cluster size, which we first show provides stable information about palatability in brief-access trials) within single estrous phases identified through vaginal cytology. The brevity of the sessions and small amounts of fluid ingested allowed us to perform this evaluation while avoiding confounds of satiation and postingestive effects.

Our results exposed phase-specific modulations of palatable sucrose and aversive citric acid preference that were roughly “mirror-images” of one another—lick cluster size was highest during metestrus for the former and lowest during metestrus for the latter. This patterning was reliable: the across-cycle preference patterns to all three proffered palatable tastes were significantly correlated, whereas correlations between palatable and aversive tastes were significantly negative. These results suggest that estrous phase directly impacts taste palatability, a centrally computed variable (Katz et al., 2001; Sadacca et al., 2012; Mukherjee et al., 2019), rather than acting on some physical/chemical property of particular tastes, and motivated further comparisons of responses to palatable and aversive tastes. These further analyses confirmed the effect to be a wholistic magnification of the range of palatabilities.

Palatability modulations linked to estrous phase can be reasonably argued to implicate lateral hypothalamic (LH) estradiol binding: among the many brain regions rich in estrogen receptors (ERs; Santollo and Daniels, 2015a,b), LH stands out for being involved in both consumption (Touzani and Velley, 1990; Fujisawa et al., 2001) and palatability processing (Li et al., 2013); furthermore, LH is directly connected to both taste and reward circuits (Fadel and Deutch, 2002; Berthoud and Münzberg, 2011) that are also involved in determining food consumption levels (Petrovich et al., 2005; Tyree and de Lecea, 2017; Fu et al., 2019). We therefore repeated our experiment while inhibiting hypothalamic ERs, a manipulation that did block the metestrus effect but failed to render palatability “cycle free,” instead delaying palatability magnification to diestrus.

Together, these results demonstrate an estrous phase-specific taste palatability modulation that is partly—but only partly—driven by hypothalamic estradiol activity. They have implications for human physiology and behavior (see Discussion).

## Materials and Methods

### Animals

Subjects were 25 female Long–Evans rats purchased from Charles River Laboratories and housed individually from the day of their arrival until the conclusion of the experiment. Rats were kept on a 12 h light/dark cycle (lights on at 08:00), given 7 d to acclimate to their new environment with *ad libitum* access to food and water before starting experiments and placed on restricted water access (15 ml every 24 h) to promote sampling behavior during experiments. Their weights, which ranged from 250 to 320 g on the first day of experimentation, were monitored throughout the duration of experimentation to evaluate basic health; each rat was handled each day to ensure that they were familiar with the researcher. All animal procedures were reviewed and approved by the Institutional Animal Care and Use Committee (IACUC) of the University.

### Equipment

Dissecting ways in which consumption of both palatable and aversive tastes change across individual estrous phases is a nontrivial problem because rodents have brief cycles—phases last, on average, 1 d. In the current experiments we, as others have done before us (Parker et al., 2002; Atchley et al., 2005), overcame this challenge through use of the Brief Access Test (BAT), which allows researchers to quantify licking to multiple tastants within a single test session (lasting ∼30–60 min) at spouts available for only 10 s at a time (Sclafani, 2006; Caras et al., 2008), thereby allowing quick assessments of behavior to a battery of tastes during each of the four estrous phases (see below for description of licking analysis and the Results section for comparison of estimates drawn from 10 s trials to those drawn from 15 min of continuous access).

The BAT apparatus (Med Associates), often referred to as “the Davis Rig,” comprises a clear plastic cage attached to a movable conveyor holding multiple drinking bottles (each containing one taste stimulus). Rats access these bottles one at a time via an oval hole in the center of the front panel of the cage that is blocked by a shutter during intertrial intervals. Prior to each exposure, the lick spout of one bottle is moved in front of the hole, such that the opening of the shutter allows the rat to lick from that taste stimulus for 10 s (the timer was started by the first lick). Individual licks complete a (low-current) circuit registered by the Windows PC controlling the process.

### Taste stimuli

To investigate whether the estrous cycle modulates taste palatability, we tested whether rats would simultaneously increase lick cluster size for multiple, chemically distinct hedonically positive tastes and decrease cluster size for multiple, chemically distinct hedonically negative tastes during particular phase(s) of the estrous cycle. Accordingly, we chose a battery including multiple palatable and multiple aversive tastes: hedonically positive (palatable) tastants included 0.3 M sucrose (caloric and sweet), 0.005 M saccharin (noncaloric and sweet), and 0.1 M sodium chloride (NaCl, salty); hedonically negative (aversive) tastants included 0.1 M citric acid (sour) and 0.001 M quinine dissolved in trace amounts of hydrochloric acid (QHCl, bitter). Water was also included in the taste battery, so that we could evaluate possible fluctuations of motivation across the estrous cycles during experimentation.

The multiple tastes within each valence category were specifically chosen to be, in all regards other than centrally computed hedonics, very different from one another. They differ in the receptor systems used to transduce them and in the neurons and circuits that are most responsive to them, for instance (Kinnamon, 1996; Cannon et al., 2005; Tandon et al., 2012). This was done in order to provide a rigorous test of whether results reflect taste hedonics: any phase-specific effects that can be accounted for in terms of properties of tastes other than palatability should be specific to particular tastes; if an observed pattern is general to all palatable tastes, and the opposite pattern is observed in both aversive tastes, by far the most reasonable interpretation will be a direct impact of cycle on central calculation of palatability.

Taste concentrations were chosen to be consistent with our earlier research which allowed insight into their respective palatabilities (Sadacca et al., 2012; Monk et al., 2014). All were purchased from Thermo Fisher Scientific with ACS Grade (save saccharin, which was FCC/USP Grade). Each was dissolved and mixed in deionized water, generated in a Millipore Direct-Q and made fresh the morning of each test day.

### Vaginal cytology and microscopy

Samples were acquired by gently grasping and lifting the animal's tail, exposing the ventrum and allowing the experimenter access to the entrance of the vaginal canal with a transfer pipette filled with room-temperature 0.1% PBS. The PBS was gently expressed, recollected, and placed in well plates. Two such samples were collected per rat, per session, and left to set for 20 min, after which excess PBS was removed. Vaginal cells in the wells were then stained using 250 µl of a 0.001 M DAPI solution left on for 5 min, perfused with 250 µl of fresh PBS, and allowed to settle for another 20 min before imaging.

Images of plated vaginal cells were obtained using a bright-field overlain with a blue fluorescence filter; this allowed visualization of the number of nucleated cells in the sample. Images were captured using both 10× and 40× magnification, in order to visualize the specific cell types (40×) as well as their distribution (10×) in the sample. Estrous cycle phase was estimated by two trained coders, based on previously determined ratios of three types of cells: leukocytes, cornified, and nucleated (Cora et al., 2015). Coders were blind to both the behavioral data and the judgment of the other coder.

Transitions between estrous phases typically happen during the dark period of the day/night cycle (Becker et al., 2005). We therefore designed our experiments to ensure that the measured estrous phase was current at the time that consummatory behavior was assayed: by consistently beginning behavioral experiments 1 h into the light cycle, and performing vaginal lavages immediately following, the results of cytology serve as a valid indication of the estrous phase during the data collection immediately preceding sample collection (Eckel and Geary, 1999). Furthermore, by performing vaginal lavages immediately following habituation and testing sessions, we were able to avoid causing the rats undue stress prior to collecting assays of behavior.

### Experimental design

Table 1 summarizes the protocol for our examination of palatability across phases of the estrous cycle. Experiments (which, as noted above, were run between 9:00 A.M. and 1:00 P.M. each day, depending on the position of the individual rat within the cohort) started with 5 d of habituation to the BAT rig. On Habituation Day 1, rats were placed in the rig for 30 min with the shutter closed, so that they could grow accustomed to the testing space. On Day 2, the shutter was open for the entire 30 mins, allowing *ad libitum* access to a spout with water. On Day 3, access to water (i.e., shutter open time) was extended to 1 h. On Days 4 and 5, the shutter was only open only briefly: 10 s trials started with the first lick; if the rat failed to lick for 60 s, the shutter dropped. Shutter closing was followed by an intertrial interval (ITI) of 30 s.

**Table 1.** Summary of experimental designs for Experiments 1 and 2

			Days: 1–5	Days: 6–10	Days: 11–15
Experiment 1	Estrous phase and behavior		Habituation + Lick Training	Tastes	
Experiment 2	LH estradiol and behavior	Group A	Habituation + Lick Training	DMSO + Tastes	ICI + Tastes
		Group B		ICI + Tastes	DMSO + Tastes

LH stands for lateral hypothalamus. ICI (80 nM of ICI 182,780 hydrochloride) is a potent α and β estrogen receptor antagonist used to block LH estrogen activity. DMSO (dimethyl sulfoxide) is the solvent for ICI.

For palatability testing, which followed this progressive habituation process, rats underwent 5 sessions (1/day) that were identical to Days 4 and 5 of habituation (e.g., lick spout available for 10 s following first lick, followed by a 30 s ITI), but sampling the 6-taste battery rather than just water. Each session consisted of 8 blocks of 6 trials; all 6 tastants were delivered (in randomized order) in each block (i.e., “sampling without replacement”). Lick and taste delivery data were saved to PC.

Once a session was complete, vaginal smears were collected from the female rats, and rats were returned to their home cages. The rig and bottles were cleaned after each session.

### Lick microstructure analysis

Licking at a spout is a reliably rhythmic behavior occurring at ∼8 Hz in rats. Researchers analyzing BAT data often use number of licks per trial (in relation to water licks) to estimate taste palatability (Dotson and Spector, 2005; Samuelsen and Fontanini, 2017). More sensitive measures are available in these data; however, these rhythmic licks cluster into relatively discrete bouts separated by pauses larger than (at least ≥2×) the normal interlick interval (Davis and Smith, 1992; Davis, 1996); the average number of licks in a bout (“lick cluster size” or simply “cluster size”) has been shown to be a more reliable marker of preference/palatability than lick counts or fluid volume—less influenced by motivational states that can confound palatability evaluation (Davis and Perez, 1993; Smith, 2001; Verharen et al., 2019; Naneix et al., 2020). Larger cluster sizes equate to higher palatability and smaller cluster sizes equate to lower palatability (and even aversiveness; Spector et al., 1998; Dwyer, 2012; Lin et al., 2014; Monk et al., 2014).

While much of the data supporting this conclusion has come from studies that offered rats relatively long periods of *ad libitum* access (minutes), even with the brief-access test we (Monk et al., 2014) and other labs (Schiff et al., 2023) have found that cluster size is well correlated with the palatability of tastes (see below for our direct comparison of these two versions of the paradigm). We therefore chose to use this variable as an operationalization of the hedonic value of the tastes used in BAT. Taking potential individual differences into account, we set the intercluster pause criterion to 0.5 s, which is standard in the field (Davis and Smith, 1992; Davis, 1996; Spector and St. John, 1998; Lin et al., 2014).

For some analyses and presentations, in order to facilitate comparisons between tastants that drive vastly different cluster sizes (e.g., to sucrose vs quinine), we normalized each animal's data using the following equation:

$$\mathrm{Normalized}\;\mathrm{Cluster}\;\mathrm{Size}=\frac{\mathrm{Cluste}r_{t}}{\;\bar{x}\mathrm{Cluste}r_{T}},\;\;$$

where Cluster*_t_* represents the cluster size occurring during a trial of a given taste and *x̅*Cluster*_T_* is the mean cluster size for that taste across all trials in all estrous phases*.* Normalizing in this manner allowed us to more directly compare how licking performance differs across estrous phase in tastants of opposite valences, thereby simplifying visualization of the valence-specific impact of sex hormones on licking.

Note that this normalization technique can exert an asymmetric effect on changes in licking—that is, factor increases are not symmetric with factor decreases (e.g., a twofold increase yields a value of 2, whereas a twofold decrease yields a value of 0.5)—and can therefore produce a skewed representation of the effect. To provide a convergent test that sidesteps this concern, we repeated the analysis using the log10-transformed normalization scores to determine whether any of the results reported above were artifacts of the normalization procedure.

### Comparison of licking behavior between short- and long-access licking sessions

As noted above, most previous studies using lick cluster analysis have involved 10 min or longer time of continuous fluid availability (Kent et al., 2002; Dwyer, 2008; Ahmed et al., 2017, but see Monk et al., 2014; Schiff et al., 2023). To test whether similar results can be gleaned from brief-access data (which is a requirement for our experiment, in that we wish to compare preferences for multiple tastes simultaneously), we performed a direct, within-rat comparison of the two paradigms.

A set of naive, water-restricted rats (*N* = 7) underwent standardized licking training in the lickometer apparatus for 5 consecutive days. Once stable licking behavior was established, the rats proceeded to a 9 d testing period consisting of three BAT sessions and six long-access test (LAT) sessions in the following order: BAT1, LAT1 (taste 1), LAT2 (taste 2), LAT3 (taste 3), BAT2, LAT4 (taste 3), LAT5 (taste 2), LAT6 (taste 1), and finally BAT3. During each BAT session, rats received ten 10 s trials of each taste. In each LAT session, rats were allowed to freely consume a single taste for 15 uninterrupted minutes, with the order of taste presentation counterbalanced across animals. A reduced battery of three taste stimuli was used in these experiments (only one taste could be delivered per day in the LAT, and performance of statistical tests required 2 LAT sessions per taste)—0.1 M NaCl (taste 1), 0.02 M citric acid (taste 2) and 0.2 M sucrose (taste 3), presented in a pseudorandom order (i.e., counterbalanced with block design) within each BAT session and across six LAT sessions. Lick timing was recorded for offline analysis.

### Intracranial cannula implantation (for LH infusions)

Following 5 d of handling, a new squad of rats (*N* = 5) underwent surgical implantation of custom-designed guide cannulas (Plastics One) into LH. Each rat was initially anesthetized with isoflurane (5%) followed by an intraperitoneal injection of a ketamine/xylazine mixture (100/5 mg/kg). Periodic intraperitoneal injections of the ketamine/xylazine cocktail (25% of the induction dose) were used to maintain stable anesthesia throughout the surgery. The animal’s head was shaved and secured in a stereotaxic frame using atraumatic ear bars, after which the skull was exposed and leveled. Support screws were attached to the skull, and bilateral craniotomies were drilled 2.8 mm posterior to bregma and 2.3 mm lateral to the midline. This placement was chosen so that the infusion cannulae could be implanted ∼0.25 mm lateral to the LH without directly penetrating it. In a pilot experiment (*N* = 5), direct penetration of the LH starkly altered behavior even during control DMSO infusions. By positioning the infusion site lateral to the LH, we were able to influence the structure pharmacologically while minimizing mechanical damage caused by cannula insertion. As observed in our pilot experiment, such damage could produce unintended alterations in consumption and animal welfare (Morgane, 1961). Cell dye (Vybrant DiI Cell Labeling Solution, Thermo Fisher Scientific) was applied to the tips of the guide cannulae (to aid with later histological identification of tip location), which were then slowly lowered to a depth of 6.1 mm (ultimately, the infusion cannulae extended 2 mm past the tip of the guide cannulae, resulting in an infusion depth of 8.1 mm) below dura. Silicone was used to seal any gaps between cannulae and skull, dummy cannulae were inserted to keep the guide clear, and dental cement was used to fix the entire assembly in place. A dust cap was placed over the cannula to prevent obstruction/infection, and rats were given postoperative injections of lactated ringer, penicillin, and meloxicam.

### Awake intracranial (LH) infusions

Infusate was either 80 nM of ICI_182,780_ hydrochloride (ICI; Sigma-Aldrich), a potent α and β estrogen receptor (ER) antagonist dissolved in 5% dimethyl sulfoxide (DMSO; Sigma-Aldrich), or the DMSO vehicle alone. Each infusate was delivered for five consecutive sessions, for a total of 10 consecutive sessions comprised of infusions and behavioral testing; infusate order was counterbalanced and found to have no impact on performance (see Results).

Infusions were performed just prior to behavioral testing sessions. The rat was cradled in the experimenter's lap, and infusate was delivered (total bilateral volume, 1.0 µl, delivered through cannulae threaded with 10 µl Hamilton syringes) over a 2 min period. Following the infusion, the infusion cannula was left in place for another minute to allow for complete diffusion of the infusate, after which the rat was returned to its home cage for 20 min to provide time for binding of ICI to receptors (de Arruda Camargo et al., 2000), and then moved to the BAT rig for a testing session identical to those described above. Every session was followed by vaginal smear collection.

### Statistical analysis

All statistical analyses [ANOVAs, *t* tests, Pearson's correlations, and chi-squared (*χ^2^*) frequency analysis] were performed using custom Python scripts, with significance criteria (alpha) set to 0.05.

As shown in Table 2, we did not have precisely the same numbers of rats in each phase in all experiments, because it was not always possible to definitively identify estrous phase; in such situations, the data were analyzed with generalized linear mixed model (GLMM) to control for the possible impact of using different numbers of datapoints in different groups (phases). The model is defined as *y* *=* *Xβ_i_* *+* *Zu_j_,* where *X* (e.g., estrous phases, taste solutions, taste valences) and *Z* (rats) are the fixed and random effects, respectively, and *β* and *u* are their coefficients. Any significant main effects or interactions revealed by these ANOVAs were followed by post hoc Tukey tests. In later analyses, the five tastes were collapsed into two groups according to palatability valence, in order to provide rigorous tests of our central hypothesis.

**Table 2.** Number of rats across phases in Experiments 1 and 2

		Estrous phases			
		Estrus	Metestrus	Diestrus	Proestrus
Experiment 1		13	7	13	9
Experiment 2	DMSO	4	5	5	4
	ICI	5	5	4	3

ICI (80 nM of ICI 182,780 hydrochloride) is a potent *α* and *β* estrogen receptor antagonist; DMSO (dimethyl sulfoxide) is the solvent for ICI.

Trials in which rats did not lick were removed from the analyses.

### Histology

After the conclusion of experimental protocols, rats were deeply anesthetized with a ketamine/xylazine mixture (200/10 mg/kg) and exsanguinated via cardial perfusions of 0.9% saline and 10% formalin. Brains were extracted and fixed in a 30%/10% sucrose/formalin solution for 2 d. After the brains were fixed, coronal slices (60 µm) were made, mounted onto slides, and stained with NeuroTrace (500/525 Green Fluorescent Nissl Stain, Thermo Fisher Scientific) to allow assessment of infusion cannula placements.

## Results

### Female rats progress through recognizable sequences of estrous phases

**Figure 1.**
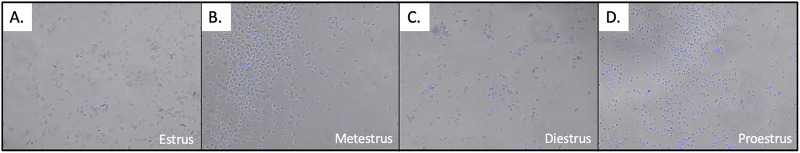
Photomicrographs of DAPI-stained Long–Evans rat vaginal smears obtained from four consecutive test days. Images are in order of estrous cycle with presence of estradiol increasing from left to right: ***A***, Estrus; classic cornified cells. ***B***, Metestrus; presence of a combination of cornified, nucleated, and leukocytes. ***C***, Diestrus; leukocytes appear in combination with nucleated cells, and ***D***, Proestrus; majority of nucleated cells appear in clumps. Note. The inter-rater congruence for identifying estrous phase; overall Cohen's κ confirmed substantial inter-rater reliability (κ = 0.672).

We evaluated estrous phase by quantifying distinct, specific ratios of cell types (Cora et al., 2015) present in photomicrographs of plated vaginal smears (Fig. 1). This visual quantification was performed by a pair of raters, each blind to both the behavioral data and the other rater's assessments. The inter-rater congruence for identifying estrous phase averaged 76.4%; agreement between the two raters' assessments as evaluated using Cohen's Kappa was in the “acceptably high” range (*κ* = 0.672; McHugh, 2012). Data from rats that experienced an irregular cycle, or for which there was disagreement on phase, were removed from analyses.

### Lick cluster size analyses of 10 s licking trials provides reliable palatability estimates

In order to test our hypotheses about each of the four phases of a 4 d cycle, it was necessary that we extract reliable estimates of the palatability of multiple tastes from single tasting sessions in which the rat consumes only small amounts of fluid. The most stable estimates of palatability have been suggested to come not from raw measurement of consumption (which tends to be influenced by several factors other than palatability; see Davis and Smith, 1992; Davis, 1999) but from calculations (and between-taste comparisons) of average lick cluster size. We therefore calculated lick cluster sizes from data collected in the brief access test (BAT). By limiting trials to 10 s, it is possible to collect repeated samples of licking to each taste while avoiding confounds arising from satiation and other postingestive effects—that is, the BAT isolates effects related to stimulus taste/palatability from those related to the impacts of stimulus ingestion.

But while a small set of papers have reported reliable palatability estimates from lick cluster analyses of BAT data (Monk et al., 2014; Schiff et al., 2023), it is far more common to calculate average cluster sizes across 15 min of bottle availability. It was therefore important to begin by comparing the results of this analysis brought to bear on the two types of session on a within-rat basis, in order to directly test whether the BAT procedure provides usable palatability information via cluster size measurement.

**Figure 2.**
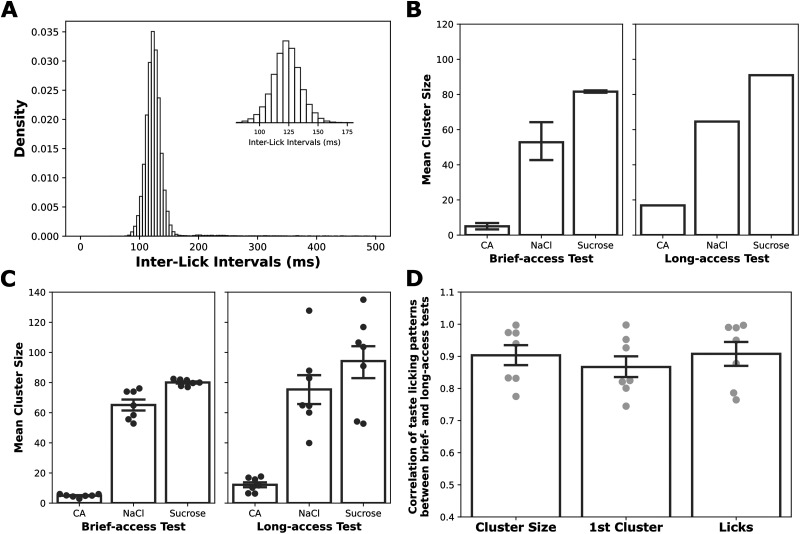
Lick cluster size analysis in the brief-access test provides a reliable estimate of palatability. ***A***, The histogram of interlick intervals from a representative rat is normally distributed with a mean of ∼125 ms, demonstrating stereotypical rodent licking behavior (lick rate ∼8 Hz); data from the entire group are shown in inlay. ***B***, Mean cluster size calculated from a representative rat’s licking performance during the brief-access test and the long-access test. Lick cluster sizes, and more importantly the relationships among cluster sizes to different tastes, were comparable across test durations. Taste stimuli and concentrations: citric acid (CA; 0.02 M), NaCl (0.1 M), and sucrose (0.2 M). ***C***, Bar plots (mean ± SEM) show the average cluster sizes across all subjects during the brief- and long-access tests for CA, NaCl, and sucrose. Dots represent individual rats. ***D***, Pearson’s correlation coefficients between licking variables measured in the brief-access and long-access tests. All three variables from the brief-access trials—mean cluster size (Cluster Size), cluster size of the first initiated cluster (1st Cluster), and average licks per trial (Licks)—were highly correlated with the mean cluster size from the long-access test sessions (Pearson's *r* ≈ 0.9). Error bars represent SEMs.

As can be seen in Figure 2, analysis of the data from such a comparison (*N* = 15 rats) supports this hypothesis. Figure 2*A* shows the tight distribution of interlick intervals (ILIs) making up the BAT data, which reliably reflected the expected ∼8 Hz lick rhythm—a mean of ∼120 ms (precise frequency = 8.33 Hz), with 95% of the values falling within 40 ms of that mean. Based on this distribution, we defined the ends of lick clusters as occurring just before ILIs >500 ms—more than twice of the average ILI. This definition minimizes the impact of occasional missed licks, allows easy identification of lick clusters, and maintains consistency with previous studies (Davis and Smith, 1992; Dwyer, 2012; Lin et al., 2014).

Figure 2*B* presents the average lick cluster sizes from a representative rat exposed to a three-taste battery (NaCl, citric acid, sucrose) in both BAT (Fig. 2*B*, left) and LAT (Fig. 2*B*, right) sessions. The rat exhibited a consistent palatability ranking across both datasets (sucrose > NaCl > citric acid); even the shapes of the distributions—sucrose somewhat higher than NaCl, NaCl several times higher than citric acid—was conserved across the long- and brief-access protocols. Note that the multitrial nature of the BAT data allows us to calculate standard error bars around the mean, which is not possible with fewer than three LAT sessions per tastant. This, in turn, enables statistical analysis—specifically, a one-way ANOVA—which revealed a significant main effect of Taste (*F*_(2,23)_ = 21.82, *p* < 0.001). Post hoc Tukey tests further showed that cluster sizes for the two palatable tastes (sucrose and NaCl) differed significantly from each other (*p* < 0.05) and that the cluster size for citric acid was, unsurprisingly, significantly smaller than those of the palatable tastes (*ps* < 0.05).

These results were representative of those obtained when averaging across all rats in the experiment (Fig. 2*C*). A two-way ANOVA revealed a significant main effect of Taste (*F*_(2,12)_ = 96.14, *p* < 0.001), but neither effect involving Trial Type reached significance (*F*_trial type_(1,6) = 3.27, *p* > 0.05; *F*_Taste × Trial Type interaction_ < 1). Post hoc comparisons following the significant Taste main effect showed that sucrose cluster sizes were greater than those for NaCl (*p* < 0.05), and citric acid cluster sizes were significantly smaller than those for both palatable stimuli (*ps* < 0.001). Note that the variability of cluster size is larger with 15 min *ad libitum* delivery, a fact that highlights the value of the BAT: the longer taste availability (and resultant increase in overall consumption) allows extraneous postingestive variables such as satiation to add noise to the data; this pattern of results thus confirms our argument that the brief-access design (and in particular the need to water-restrict rats for the BAT) does not impose a ceiling effect on licking of palatable tastes and that cluster size analysis brought to bear on BAT data permits detection of even subtle differences in palatability.

Of course, the averaged data could show overall high levels of preference for sucrose and low for citric acid in both paradigms despite differences in what tastes individual rats prefer in each paradigm (Fig. 2*C* could hide the fact, for instance, that one rat prefers sucrose in the BAT and NaCl in *ad libitum* consumption while a 2nd rat does the opposite). We therefore performed a statistical assessment of the Pearson's correlation value (*r*) between BAT and LAT data across all rats. This analysis applied to cluster sizes (Fig. 2*D*, left bar) revealed high similarity (*r *≈ 0.9) between the two trial types. When the analysis was restricted to the first lick cluster in a trial (Fig. 2*D*, middle bar) of BAT (as has also been used to evaluate palatabilities), the correlation was still high, but slightly lower. Finally, and as expected, the between-taste differences in number of licks per trial in the BAT session (Fig. 2*D*, right bar) were also well correlated to the average cluster size in the LAT sessions.

These findings confirm that cluster size is a sensitive and reliable measure of taste palatability using BAT data (and the fact that those correlations are <1.0 likely reflects phase-specific effects investigated below). Given the BAT's additional felicitous properties (the fact that BAT allows evaluation of licking of multiple tastes in the same short time period, and the fact that BAT results cleanly reflect taste palatability without confounding influence of postingestive factors), we conclude that these data provide a valid measure of palatabilities across an entire taste battery in single sessions, clearing the way for its use to estimate taste palatability in different phases of the estrous cycle.

### Estrous phase acts similarly on tastes of similar palatability

**Figure 3.**
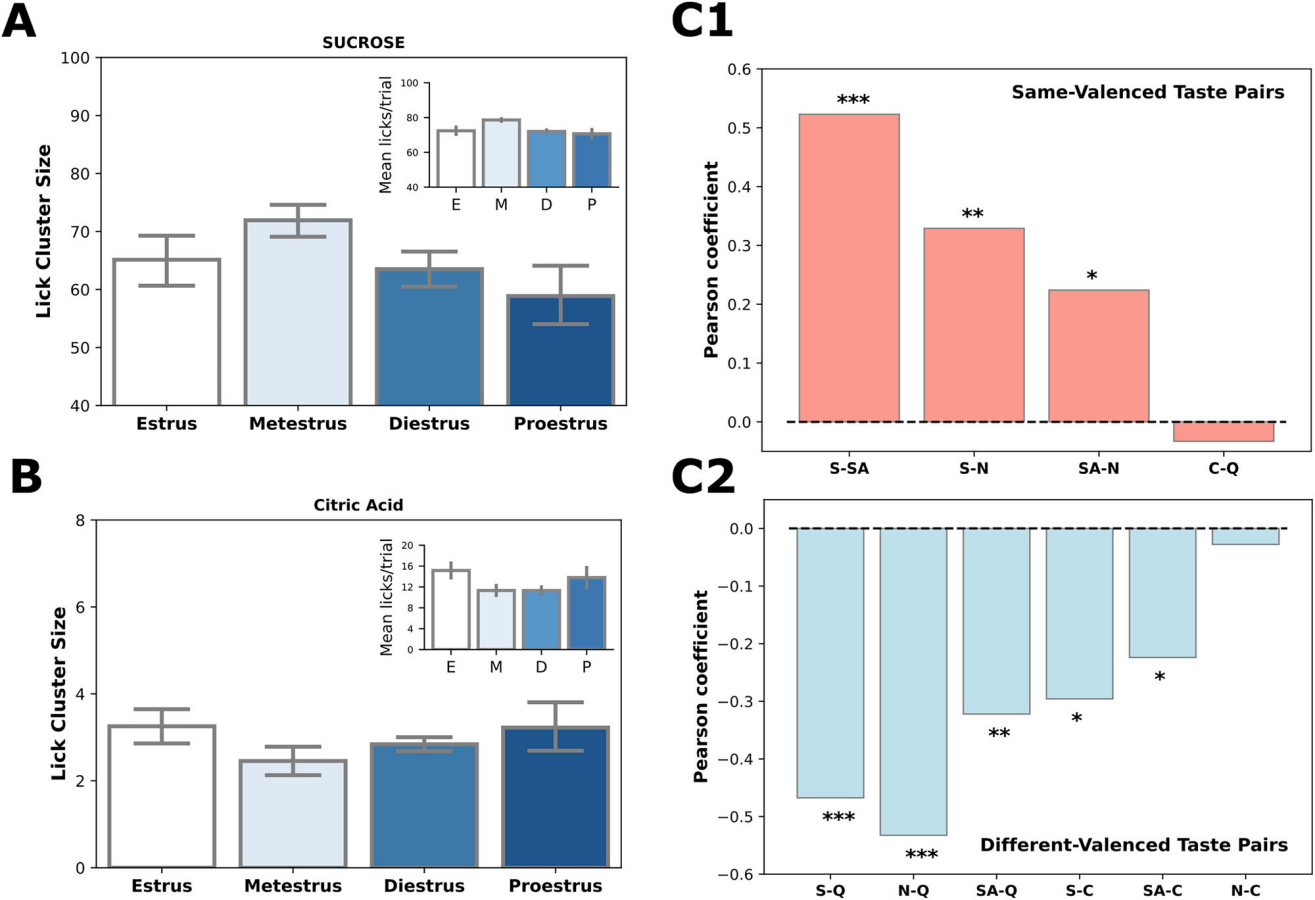
Estrous phase impacts palatability similarly across multiple tastes with the same valence. ***A***, Lick cluster size during consumption of the palatable tastant sucrose fluctuates across estrous phases, peaking at metestrus. The inset shows the number of licks per trial for sucrose trials (E, estrus; M, metestrus; D, diestrus; P, proestrus). ***B***, Lick cluster size during consumption of the aversive tastant citric acid also fluctuates across estrous phases, with the smallest cluster size observed at metestrus. The inset shows the number of licks per trial for citric acid trials (E, estrus; M, metestrus; D, diestrus; P, proestrus). ***C***, Pearson’s correlation coefficients between cluster sizes across estrous phases for pairs of tastes with the same valence (***C1***) and for pairs of tastes with different valences (***C2***). All but one same valence pairs showed significant positive correlations, and all but one difference-valence pair showed negative correlations, suggesting that changes in taste preference across estrous phases are similar among tastes with the same valence but opposite between tastes with different valences. *p < 0.05. **p < 0.01. ***p < 0.001. Error bars represent SEM. S, sucrose; SA, saccharin; N, NaCl; C, citric acid; Q, quinine-HCl.

Bringing this central analysis to bear on sucrose (a palatable taste) trials reveals what appear in purely visual analysis to be estrous phase-specific effects, as shown in Figure 3*A*. Inspection of the figure suggests that cluster size was largest in metestrus and smallest in diestrus, which implies that rats' preference for sucrose was highest during metestrus; the same (but rougher) pattern across estrous phases could be seen in the average number of licks per trial (Fig. 3*A*, inset). Meanwhile, the opposite pattern appeared in both lick cluster size (Fig. 3*B*) and licks/trial (Fig. 3*B*, inset) for the aversive taste citric acid, which was most aversive during metestrus.

While the small subsets of data that are represented in Figure 3*A*,*B* make it impossible to go beyond visual analysis (i.e., the effects do not alone reach significance), between-taste correlations revealed the validity and reliability of the across-cycle preference patterns: when we performed pairwise correlations (using Pearson's *r*) on the pattern of lick cluster size across estrous phase for all of the tastes in all rats, the correlations between all of the pairs of palatable tastes were significantly positive (Fig. 3*C1*; the low cluster lengths for both of the aversive tastes made it impossible to reliably measure the correlation between them). Furthermore, when we compared pairs of tastes that differed in palatability (Fig. 3*C*2)—comparing sucrose to quinine, for instance (leftmost bar)—the correlations were significantly negative in all but one of the six comparisons. Thus, we can conclude that preferences for all three palatable tastes vary similarly across estrous phases and that preferences for both aversive tastes vary across estrous phase in a way that is a mirror image of the palatable pattern.

The fact that the impact of cycle phase was similar for all of the palatable tastants despite the vast differences between NaCl, saccharin, and sucrose—dissimilarities of chemical structure, transduction mechanisms, activated systems, etc.—strongly implies that estrous cycle acts, to some extent, directly on the central calculation of palatability, which is the most notable similarity between the three stimuli, rather than acting on some property of a particular taste. This conclusion is further supported by the fact that the same is true of citric acid and quinine, which share few properties other than aversiveness. In summary, licking to our taste battery depends upon estrous phase, and the defining variable that determines these licking differences is not any characteristic of particular tastes but rather (the centrally-calculated variable of) palatability.

### Between-phase differences in palatabilities reflect metestrus-specific magnification of the normal palatability range

The above results and analyses directly motivate (but fall short of testing) our central hypothesis, which is that the range of palatabilities becomes magnified during metestrus, when cluster size is particularly high for palatable tastes and particularly small for aversive tastes. Testing this hypothesis involves direct, across-cycle comparisons of tastes organized in terms of palatability (as justified by the Fig. 3 results)—that is, collapsed into palatable and aversive categories.

Given the many-fold difference in average cluster size for palatable and aversive tastes (Fig. 2*B*), however, direct comparisons of raw changes from phase to phase can be difficult to visualize and interpret. To address this, we normalized lick cluster sizes for each taste to the average across the entire estrous cycle for that specific taste prior to further analysis (see Materials and Methods). Thus normalized, between-phase differences in lick cluster size become easily comparable, and it becomes possible to analyze data from both valences together, phase-by-phase, and thereby to directly test whether the range of perceived palatability expands and contract holistically (i.e., whether increases in lick cluster size for palatable tastes is accompanied by simultaneous decreases of lick cluster size for aversive tastes) with estrous phase.

**Figure 4.**
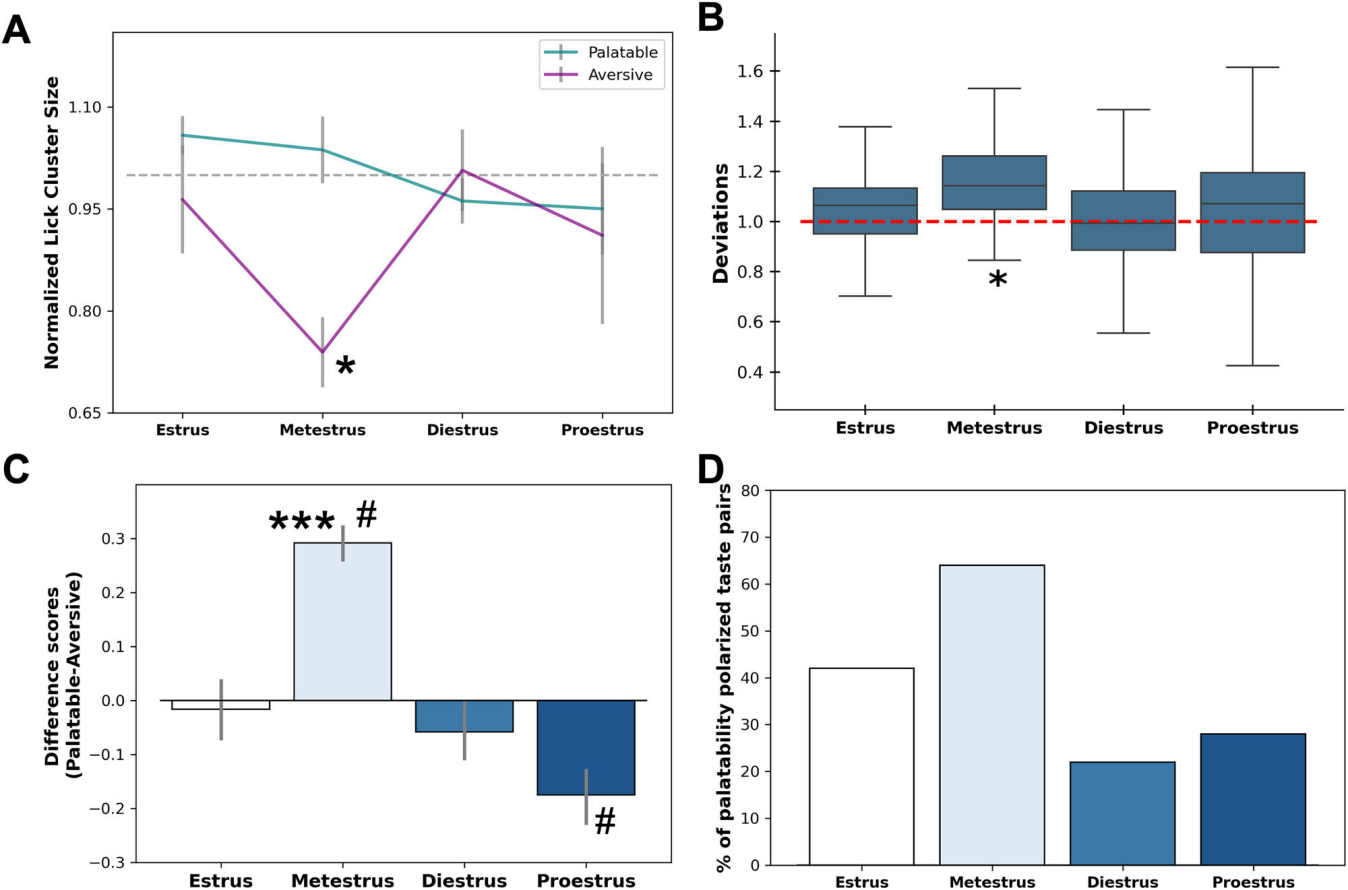
Palatability range is magnified during metestrus. ***A***, Quantified as normalized cluster size, preferences for palatable tastes are elevated during metestrus, and preferences for aversive tastes are simultaneously reduced during metestrus relative to other phases (*p < 0.05 difference between palatable and aversive tastes). ***B***, Box plot assessing the magnification of palatability, quantified as the deviation of cluster size from 1 (the null population mean), across estrous phases. Cluster size deviated most during metestrus, indicating the greatest shift in perceived palatability (*p < 0.0125, significantly different from 1). ***C***, Difference scores calculated between palatable and aversive tastes (see text for details) confirmed the amplification of taste palatability (*y*-axis) during metestrus. ***D***, Percentage of palatable-aversive trial pairs displaying palatability polarization performance across each estrous phase. ***p < 0.001 higher than other groups, ^#^*p* < 0.05 different from 0. Error bars represent SEMs.

These normalized data are presented in Figure 4*A*, which suggests, as expected, a phase-specific modulation of palatability centered primarily on metestrus. A two-way ANOVA brought to bear on these data reveals a significant palatability × phase interaction (*F*_(3,80)_ = 2.96, *p* < 0.05). It appears that lick bouts to palatable tastes were relatively high during both estrus and metestrus, but only in metestrus was normalized licking of palatable and aversive tastes distinct from one another, with palatable tastes preferred more than normal and aversive tastes disliked more than normal. This observation was confirmed by a GLMM, which showed that only during metestrus were normalized lick cluster sizes for palatable tastes significantly different from those of aversive tastes within the same phase. (*β *= 0.38, *p* < 0.05).

We moved on to perform a more conservative test on these data. We reasoned that if the range of palatabilities is magnified during metestrus, this magnification should be reflected in the degree to which normalized cluster size for both aversive and palatable tastes deviate from 1, which is the across-phase average. To test this hypothesis, we combined data from both palatable and aversive tastes after first transforming the aversive normalized lick cluster size by subtracting it from 1 (thus allowing responses to both palatable and aversive tastants to be thought of in terms of their deviation from the average) and conducted one-sample *t* tests against the null population mean (i.e., 1) for each phase, using a Bonferroni-corrected significance threshold of *p* = 0.0125.

We call this a conservative test of phase specificity because the data are biased toward being >1 for multiple phases. Despite this fact, and consistent with our prediction, a significant deviation was observed only during metestrus (*t* = 4.83, *p* < 0.001; Fig. 4*B*). Together, these findings (which were replicated using log-transformed data, thereby controlling for asymmetries in the original normalization; data not shown) provide strong evidence that hormonal fluctuations across the estrous cycle drive expansion and contraction of the perceived palatability range.

To further test the robustness of this conclusion, we generated an unbiased estimate of the phase specificity of palatability by randomly sampling 50 pairs of trials between the two valences of tastes in each cycle phase and calculating the mean differences between the pairs. Repeated 100 times, this bootstrapping process offered a direct estimate of relative magnitudes of the spread of palatability within each estrous phase. As shown in Figure 4*C*, this analysis revealed a significant effect (*F*_(3,396)_ = 64.64, *p* < 0.001), indicating a magnification of the difference between palatable and aversive tastants during metestrus (*p* < 0.05). Additional one-sample *t* tests (against a null population mean of 0) confirmed a robust magnification during metestrus (*t* = 18.70, *p* < 0.001) and a mild diminution during proestrus (*t* = −5.55, *p* < 0.05). No significant magnification or diminution was observed during estrus (*t* < 1) or diestrus (*t* = −1.33, *p* > 0.05). In every phase, palatable tastes were hedonically positive and aversive tastes were hedonically negative, but the difference between the two was at its most extreme by far during metestrus (1–2 d following the plasma estradiol peak).

We performed one final convergent test of our hypothesis, analyzing the entire cross-phase distribution using *χ^2^* (an analysis that requires that the data be in the form of integers). For all palatable-aversive trial pairs from each session, we compared the differences between the cluster size in those trial pairs to the average difference for that rat across all sessions. The results of this analysis are shown in Figure 4*D*, plotted in terms of the percentage of trials in which the polarization of the two palatability extremes was larger than the average preference difference. The distribution was found to be significantly nonuniform (*χ*^2^_(3)_ = 21.94, *p* < 0.001), with the percentage of trials in which palatable-aversive polarization exceeded the overall average topping 50% during metestrus only. We therefore, on the basis of this and the above tests, conclude that the natural range of palatabilities is magnified while female rats are in metestrus.

Of course, other nonhormonal factors are known to impact perceived palatability across a series of tasting sessions. Most notably, the attenuation of neophobia (the fear of new tastes) is expected to increase preferences for tastes as they become familiar (Lin et al., 2012; Monk et al., 2014; Menchén-Márquez et al., 2026). Given that we were not able to counterbalance order of sessions (because each phase is of different duration), any novelty effect is not spread evenly across phase—more often than not (i.e., in 71% of the rats), the first tasting session occurred during the diestrus phase. It is therefore possible that the apparent metestrus effect is actually a familiarity effect in disguise.

**Figure 5.**
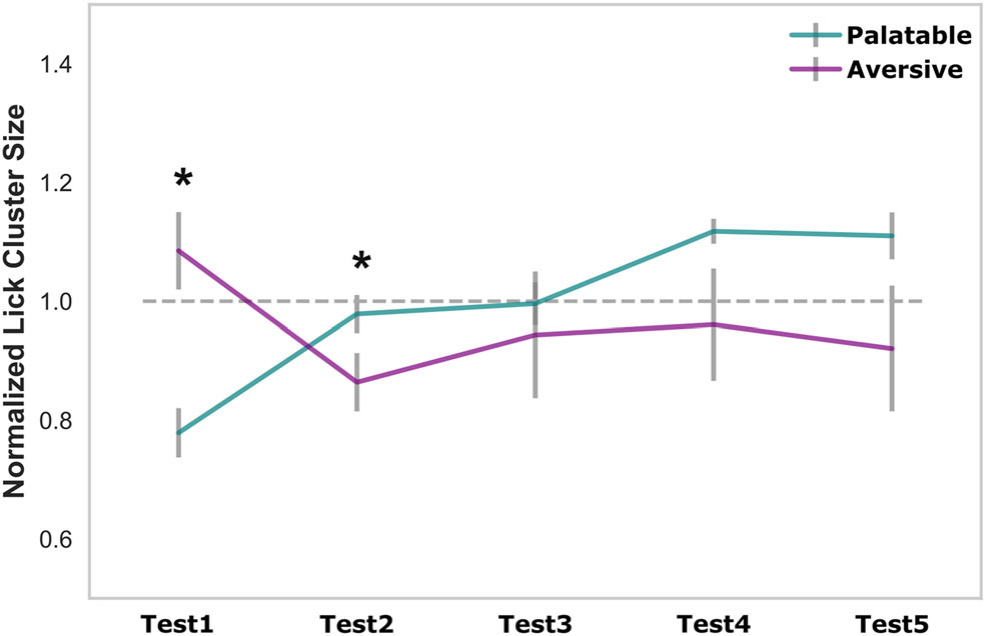
Taste preference varies across sessions ordered by testing sequence. Mean (±SEM) normalized cluster size for palatable and aversive tastes across testing sessions ordered by test sequence. A two-way repeated-measures ANOVA revealed a significant Valence × Session interaction (*F*_(4,44)_ = 3.17, *p* < 0.05), reflecting an increase in normalized cluster size from Test 1 to Test 2 for palatable tastes and a decrease over the same interval for aversive tastes (*ps* < 0.05). Preferences then stabilized from Test 2 through Test 5.

We tested this possibility by reorganizing our data according to order (Fig. 5), a presentation that clearly revealed a novelty effect. Specifically, palatable tastes tend to be less palatable for female rats in the initial session, as predicted by work on neophobia. A two-way ANOVA revealed a significant Test × Valence interaction (*F*_(4,44)_ = 3.17, *p* < 0.05), whereas neither the main effect of Test (*F* < 1) nor Valence (*F*_(1,11)_ = 3.45, *p* = 0.09) reached significance. Post hoc comparisons showed that palatable tastes exhibited increased cluster sizes from Test 1 to Test 2 (*p* < 0.05), whereas aversive tastes showed decreased lick cluster sizes over the same period (*p* < 0.05); cluster sizes then stabilized, with no significant changes from Test 2 to Test 5 (*ps* > 0.05). Notably, however, this effect is in the opposite direction to that observed in metestrus, and later sessions are all essentially identical; that is, this presentation fails to explain, and in fact obscures the metestrus effect—the single estrous phase in which both palatable tastes are more preferred than usual and aversive tastes are less preferred than usual.

### Inhibition of hypothalamic estradiol binding reduces the metestrus “magnification” of palatability range but does not flatten the palatability spread across the cycle

Estradiol is the reproductive hormone most commonly implicated in feeding and related processes (Wade and Gray, 1979; Kensicki et al., 2002; Eckel, 2004; Rivera and Stincic, 2018), and thus it is reasonable to hypothesize that the action of estradiol might explain the metestrus magnification of the palatability described above. On the basis of previous results and the logic laid out by Asarian and Geary (2013) (see also Eckel, 2004), we specifically hypothesized that this effect might reflect a delayed result of estradiol-driven activity in LH, which is (1) involved in feeding (Anand and Brobeck, 1951a,b; Margules and Olds, 1962); (2) connected to other systems involved in palatability (Fadel and Deutch, 2002; Petrovich et al., 2005; Berthoud and Münzberg, 2011; Tyree and de Lecea, 2017; Fu et al., 2019); (3) rife with neurons that produce palatability-related taste responses (Li et al., 2013); and (4) rich with estrogen receptors (Laflamme et al., 1998; Rivera and Eckel, 2010; Frank et al., 2014).

To test this hypothesis, we prepared rats with bilateral guide cannulas placed just above LH (to be precise, cannulae were placed 0.1 mm lateral to the center of LH—pilot experiments made it clear that it is difficult to avoid damaging LH with direct infusions and that the more effective method was placing larger infusions nearby LH; see Materials and Methods). We then ran these rats through the same experimental protocol described above (daily preference testing followed by vaginal smears); 20 min prior to each experimental session, however, we infused either ICI_182,780_ (an estrogen α and β receptor antagonist) or DMSO (the vehicle control) into the region of LH. Using this procedure, we were able to evaluate the impact of LH estradiol inhibition on the above-described cycle-related mediation of taste palatability.

We suppressed hypothalamic estradiol binding for all sessions across one entire cycle (the fact that we were only able to identify phase after a session made experimental targeting of only individual phases impossible without running a large number of rats and accepting substantial data loss). Given the fact that free-floating estradiol levels are almost nonexistent during metestrus itself, it is a reasonable assumption that any impact of the estradiol manipulation was due to inhibition of binding during the phases characterized by high estradiol levels—that is, this experiment effectively tested whether blocking the estradiol peak during diestrus/proestrus blocked the magnification of palatability 1–2 d later. As noted elsewhere, this would be consistent with the delayed impact on consumption following either estradiol treatment (Gray and Greenwood, 1982) or estrogen receptor activation (Santollo et al., 2007).

Each rat was tested with each infusate, such that comparisons could be made within-rat and any physical impact of implantation and liquid infusion would not explain any drug-specific/phase-specific preference patterns. Furthermore, the order of drug condition was counterbalanced, and examination of which estrous phase rats were in at the start of testing revealed no bias toward any particular phase, with two rats in estrus and one rat in each of the remaining three phases; thus, the likelihood that testing order impacts our conclusions is minimal and justify inclusion of data from all testing days in the analysis. Our prediction was that ICI infusions (i.e., inhibition of estradiol binding in LH) would eliminate the palatability magnification observed in metestrus and would more generally “flatten” the cycling of palatability.

**Figure 6.**
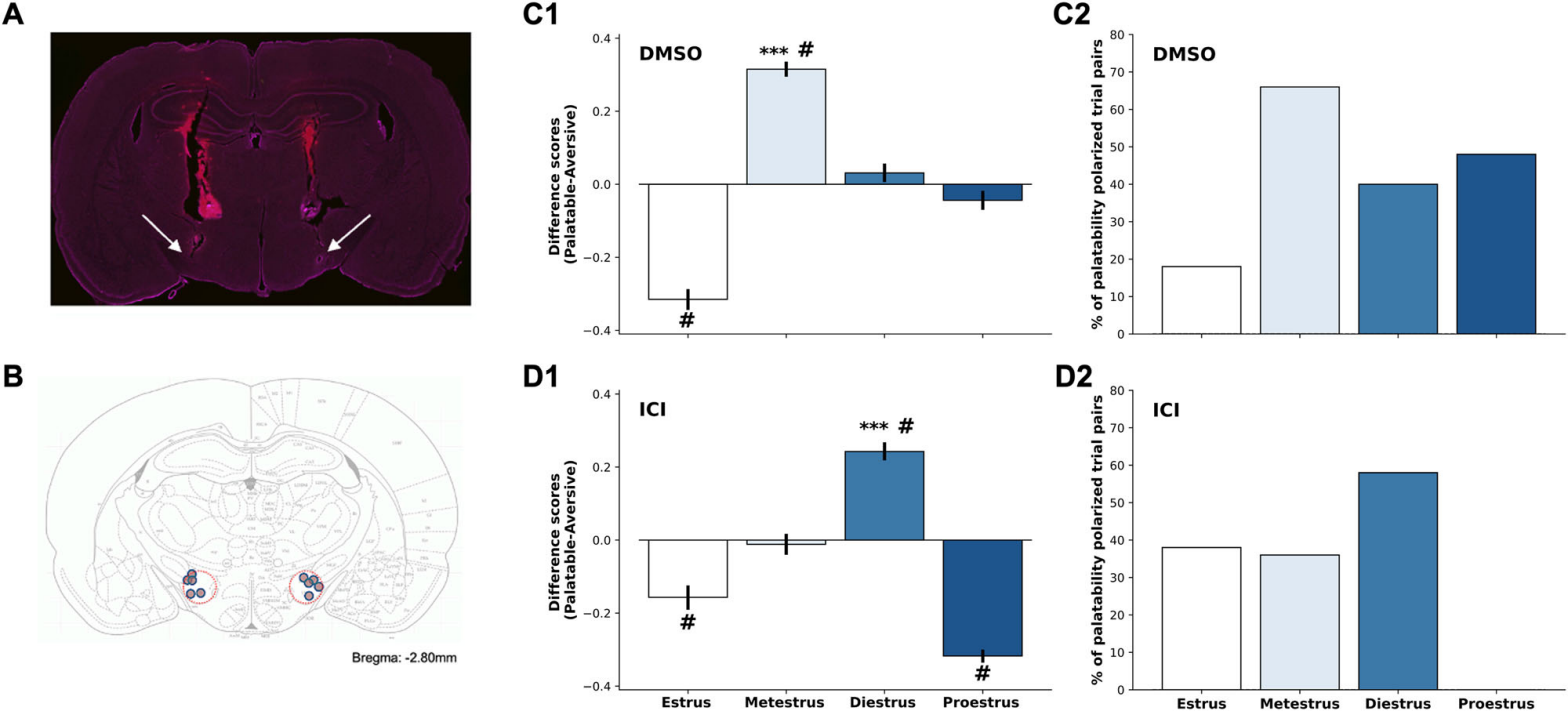
Blocking LH estrogen receptors shifts the timing of palatability preference magnification. ***A***, A representative coronal section from single animal showing the placement of guide cannula. The image of the coronal section was taken at low magnification under bright-field microscopy and show Nissl Stain (magenta), placement of cannula (red), and extension of infusion cannula (2 mm below guide cannula tips) in the LH. ***B***, Coronal illustrations of the rat lateral hypothalamic area are depicted with the area in which bilateral cannula tips were identified in pink (each dot represents the placement of a single infusion cannula for each animal in the dataset). Correct placements were spaced from bregma −2.8 mm according to the Paxinos rat brain atlas (Paxinos and Watson, 2006). ***C***, Taste palatability polarization in metestrus characterized by preference difference scores (***C1***) and palatable-aversive trial pairs (***C2***) during DMSO-infused behavioral sessions. ***D***, Taste palatability polarization in metestrus characterized by preference difference scores (***D1***) and palatable-aversive trial pairs (***D2***) during ICI-infused behavioral sessions. These ancillary analyses indicate that similar to the nonsurgical rats (Fig. 4), DMSO-treated rats exhibited the highest polarization during metestrus. Conversely, the highest polarization seemed to occur during diestrus for rats pre-treated with ICI. ***p < 0.001, indicating a significant magnification of taste preferences in metestrus than other phases; ^#^*p* < 0.05 different from 0. Error bars represent SEM.

Figure 6*A* shows a representative coronal section stained for Nissl substance and imaged using bright-field microscopy showing bilateral cannula tracks leading to the vicinity of LH. Figure 6*B* is the coronal schematic containing LH, overlain with dots marking the placements of infusion cannula tips for all five rats used in this experiment (one rat for which one infusion failed to target LH was removed prior to examination of licking data). Because an initial three-way ANOVA (drug × order × estrous group) failed to reveal significant drug order effects on cluster size for palatable or aversive tastants, we combined data across orders of infusion for the analysis of palatability patterning (again quantified in terms of normalized cluster size).

We first analyzed the control (DMSO-infusion) data, using the same pair of analyses brought to bear on data from nonsurgical rats (Fig. 4*C*,*D*). Both analyses replicated the finding of a metestrus magnification of palatability range, with the only difference from the nonsurgical results having to do with a surprisingly low palatable versus aversive difference in a different phase (estrus). While we have no conclusive explanation for this single discrepancy (although see Discussion), the bootstrapping test (compare Figs. 6*C1*, 4*C*) shows that normal preferences were magnified during metestrus compared with all other phases (*F*_(3,396)_ = 1,725.86, *p* < 0.001), and the matched trials test (compare Figs. 6*C2*, 4*D*) shows that the pattern of palatability magnification across the four estrous phases did not differ significantly between nonsurgical and DMSO datasets (*χ*^2^_(3)_ = 6.83, *p* > 0.05). These analyses confirm that the control data largely replicated the phenomenon described in Figures 3 and 4.

Inhibition of LH estrogen receptors, meanwhile, had a substantial impact on this taste preference pattern, although that impact was not as simple as we had hypothesized. The bootstrapping test (Fig. 6*D1*) revealed (as predicted) that the tests performed following infusion of ICI into LH showed a complete lack of the previously observed metestrus magnification of the lick clustering differences between palatable and aversive tastants observed in control sessions (*p* < 0.05). That is, inhibiting hypothalamic estradiol binding (presumably during diestrus when estradiol levels are high) eliminates the general magnification of palatability that normally appears 1–2 d later. Note, however, that data from cycles in which ICI infusion inhibited LH estradiol binding was far from flat: in fact, further analysis revealed a novel magnification of the normal patterning during diestrus (*F*_(3,396)_ = 697.47, *p* < 0.001). It appears that LH estradiol inhibition delays, rather than eliminating, the cycle-related modulation of taste palatability processing.

The matched pairs test (Fig. 6*D2*) confirmed these impacts of LH estrogen receptor blockade, showing that the across-phase pattern of palatability magnification for ICI-treated rats was significantly different from those of both the nonsurgical group (*χ*^2^_(3)_ = 25.71, *p* < 0.05) and the DMSO group (*χ*^2^_(3)_ = 31.55, *p* < 0.05). Note that each of these *χ*^2^ values is more than three times the size of the *χ*^2^ comparing the DMSO and nonsurgical groups, despite the several differences between the rats from which Figures 3 and 5*A* were generated (surgery, head tethering, fluid infusion, etc.), a fact that reinforces the conclusion that the metestrus phenomenon is a robust, delayed impact of the hypothalamic action of estradiol. While the fact that ICI does not flatten the pattern across the entire cycle must await explanation in future work (see Discussion), the activity of estradiol in LH clearly plays a role in central palatability processing.

Although designs involving multiple intracranial infusions, such as the one used here, are not uncommon (Perez-Leighton et al., 2012; Eghtesad et al., 2022), one could argue that repeated infusions may cause tissue damage and thereby come to influence behavior in later sessions. To rule out this potential confound, we compared licking performance during the first four sessions with that during the last four sessions, testing the hypothesis that lesion-related effects would be more pronounced in later sessions. Contrary to this prediction, we found no significant main effect of Session (*F*_(1,4)_ = 1.44, *p* > 0.05) and no significant Session × Valence interaction (*F*_(1,4)_ = 3.28, *p* > 0.05). We can therefore conclude that infusion-related tissue damage does not account for the observed phase shift in palatability magnification during ICI infusions.

### Between-phase fluctuations in water consumption cannot explain the palatability differences

The above analyses reveal that the impact of estradiol on fluid consumption is both taste and phase dependent. While these results are naturally interpreted in terms of our hypothesized phase modulation of taste palatability, we cannot ignore the potential confounding contribution of phase specificity of hydration need. We directly addressed this possibility, analyzing across-phase differences in water intake in a similar manner to the above analysis of taste stimulus consumption.

**Figure 7.**
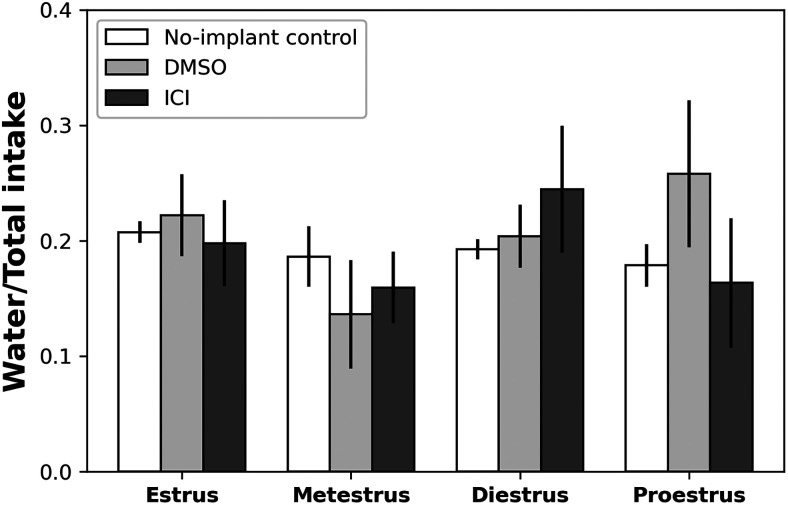
Minimal fluctuations in water consumption across estrous phases. Mean (±SEM) normalized water intake (water intake/total fluid intake) across estrous phases for nonimplant controls and animals receiving either DMSO or ICI infusions. A two-way ANOVA revealed no significant main effects (*ps* > 0.05) and no significant Group × Phase interaction (*p* > 0.05).

To control for individual differences in fluid consumption, water intake was first normalized to total fluid intake (water intake/total intake). Figure 7 shows the normalized water intake (mean ± SEM) for both nonimplanted (No-implant Control condition) and LH-cannulated rats (DMSO and ICI conditions). A two-way ANOVA revealed no significant main effect of Group (*F*_(2,99)_ = 0.22, *p* > 0.05) and no significant main effect of Phase (*F*_(2,99)_ = 1.73, *p* > 0.05); the Group × Phase interaction also did not reach significance (*F*_(6,99)_ = 1.22, *p* > 0.05). The lack of any effect of (or interaction with) estrous phase suggests that the need for fluid consumption cannot explain the observed impact of estradiol on taste palatability (see Discussion).

## Discussion

Gonadal hormones (e.g., androgens and estrogens) are primary drivers of sex differences in consummatory behavior in rats (Zucker, 1969; McGivern et al., 1996; Clarke and Ossenkopp, 1998; Asarian and Geary, 2013; Mikhail et al., 2021; for a review see Asarian and Geary, 2013). Cycle-linked fluctuations in the plasma levels of these hormones, and particularly of estradiol, have been shown to underlie consumption variability across estrous phases; in this, they mirror analogous hormone-dependent shifts in cycling human females, in whom consumption increases on the order of ∼150–170 kcal/day in the midluteal phase (compared with the midfollicular phase; Martini et al., 1994; Barr et al., 1995; Hirschberg, 2012; Vigil et al., 2022; Rogan and Black, 2023; Tucker et al., 2025).

Some research has suggested specific menstrual cycle modulation of sweet taste preference and liking (Pliner and Fleming, 1983; Frye et al., 1994; Malo-Vintimilla et al., 2024) that is mitigated by menopause (Kogo, 2003; Skoczek-Rubińska et al., 2021; Harika et al., 2024), but direct evidence for cycle-dependent shifts in human palatability processing remain scarce, just as is true for rats. To seek more definitive evidence for or against between-phase differences in palatability processing, we evaluated licking for a battery of tastes in (almost) real time, acquiring data at each phase of (almost) each rat's estrous cycle. Our analysis of cluster size revealed a general magnification of normal taste palatability patterns—the palatability range is magnified, and that magnification is largely localized to metestrus, the 1 d phase that occurs 1–2 d after the estradiol peak. Licking to palatable tastes is enhanced for both estrus and metestrus, but metestrus is the only phase in which a general palatability-related effect appears.

As to whether female sex hormones enhance palatability processing during the estrus phase or dampen palatability processing in other phases, our work is agnostic. We would speculate that both are true—a speculation supported both by the impact of LH estrogen receptor blockade, which both enhanced and depressed palatability processing (Fig. 6*D1*), and by the examination of order effects, which suggest a range of preferences that is intermediate between the normal metestrus range and other ranges (Fig. 5). We have collected data from males that is broadly consistent with this conclusion, but the many differences between male and female rats makes us loathe to conclude that the lack of female sex hormone determines their performance. Ultimately, experiments on hysterectomized females with and without hormone replacement will provide the best test of this question, although these come with caveats as well.

The magnification of palatability during metestrus aligns with previous findings showing increased food intake following the estrus phase (Ter Haar, 1972; Fantino and Brinnel, 1986); in essence, when food tastes better, consumption increases. The fact that combining hedonically similar tastes allowed us to reliably observe a significant phase-specific palatability polarization effect, despite the fact that these tastes are very different in quality, transduction mechanisms (Kinnamon, 1996; Gilbertson et al., 2001; Taruno et al., 2021), and neural systems activated (Cannon et al., 2005; Shibata et al., 2009; Tandon et al., 2012), serves as a testament to the robustness of the effect and as particularly strong evidence for our hypothesis that estrous cycle phase specifically modulates palatability directly.

We were somewhat surprised by the absence of a full palatability effect during the estrus phase (which starts ∼24 h before metestrus), although intake of each palatable taste is already high by then. This phase is typically associated with suppression of food intake (Asarian and Geary, 2002; Parker et al., 2002). If taste palatability were the sole mechanism by which gonadal hormones modulate intake, one would expect a corresponding decrease in palatability during estrus, which was not observed in the present study. It is possible that water restriction—which heightens motivation to drink—may have masked the estrus phase suppressive effect observed in earlier studies. Regardless, it is likely that estrous-related modulation of food intake is a multifactorial phenomenon and that only one of those factors is palatability.

Previous research has demonstrated that another factor playing a key role in regulating food consumption across the estrous cycle is negative postingestive feedback (Eckel, 2004). This regulation also appears to be phase specific: blocking postingestive feedback affects intake during estrus but has little impact during metestrus or diestrus (Eckel and Geary, 1999). If this is the case, it is not surprising that we observed minimal estrus-phase effects in our study given that we used the brief-access task (BAT), which is designed to isolate palatability by minimizing the impact of postingestive feedback. The mechanisms driving estrous phase changes in feeding behavior may be different for different phases.

It is also difficult to reconcile our results with those of Atchley et al. (2005), however, in which researchers used the same task employed here and showed a reduced licking of sucrose in estrus compared with diestrus (metestrus and proestrus licking were not reported). We saw no significant difference between licking in these two phases, a difference that could conceivably reflect concentration choice—the biggest estrus-diestrus difference observed by Atchley et al. was obtained with a much lower concentration of sucrose than that used here.

Another possibility is that our near-simultaneous delivery of tastes ranging from highly palatable to unpalatable in single sessions may have influenced the rats' behavior, thereby causing a difference between ours and this earlier work. As noted earlier, taste palatability is intrinsically a comparative measure—an animal's preference evaluation is dependent on the nature of the other tastes available (Flaherty and Rowan, 1986; Flaherty et al., 1995; Ballintyn et al., 2023). It would therefore be not entirely surprising to find differences in the consumption of a taste when it is delivered alone and when it is delivered among tastants of different palatabilities; the few studies (Clarke and Ossenkopp, 1998; Parker et al., 2002) that have examined cycle-dependent responses to multiple tastes have rendered results that are in broad accord with our own.

The fact that the cycle-related modulation of palatability observed here occurs during a phase with very limited estradiol circulation might suggest that a full accounting of the mechanism underlying sex hormone-dependent modulation of food consumption will be temporally and hormonally complex (see below), but on the other hand it is consistent with extensive work suggesting that the impact of even an externally induced estradiol surge in ovariectomized rodents is delayed by 24–48 h (Gray and Greenwood, 1982; Santollo et al., 2007)—a theory explicitly explored by Asarian and Geary (2013) as well as Eckel (2004), both of whom have suggested that the impact of the estradiol surge on consumption is delayed by ∼24 h. Regardless, our results jibe well with the off-cited hypothesis that food intake and taste palatability are regulated by the binding of estradiol to receptors in LH (Li et al., 2013; Santollo and Daniels, 2015a,b), in that daily intra-LH infusions of an estradiol receptor antagonist (ICI) curtailed the metestrus polarization of palatability. Given the near lack of free-floating estradiol during metestrus itself, it is reasonable to conclude that receptor blocking during the estradiol surge impacted behavior with the oft-noted 1–2 d delay.

Unpredicted, however, was the finding that inhibition of LH estrogen receptor activity appeared to delay, rather than completely eliminate, cycle-related fluctuations of palatability. While we have no definitive explanation for this result, it may reflect the fact that estradiol is only one element of a complex driver of taste palatability processing. Research has uncovered a close interaction between estradiol and cholecystokinin (CCK) in modulating food consumption (Butera et al., 1993; Asarian and Geary, 1999; Eckel et al., 2002), for instance. Specifically, the impact of CCK on feeding is critically influenced by levels of circulating estradiol, such that under conditions of normal cycling, female rat food intake is suppressed more by CCK administration during diestrus (when estradiol circulation is high) than metestrus (when estradiol circulation is minimal; Dulawa and Vanderweele, 1994). Given this diestrus-focused influence of CCK, it is possible that inhibition of LH estrogen receptor activity causes taste preference to be dominated by CCK-signaling modulation during diestrus, when estradiol levels rise. Such modulation likely occurs in regions outside the LH, such as the paraventricular nucleus of the hypothalamus (Butera et al., 1996).

Note, however, that even this description of the system almost certainly leaves out several important players. Given the complexity of both the hormonal environment and neural circuitry, palatability is likely the product of an interplay between multiple hormones also known to fluctuate across the estrous cycle (e.g., luteinizing hormone and progesterone). Thus, our findings also highlight the value of studies looking at natural fluctuations, which enhance the interpretability of ovariectomy experiments, and also the need for more complex theories regarding the regulation of food intake by sex hormones.

Finally, it is important to address the fact that intracranial infusions of DMSO had a nonzero impact on licking (compare Figs. 4*C*, 6*C1*), reducing the difference between palatable and aversive tastes (indicated by negative difference scores) during the estrus phase of the cycle. At present we are unable to say whether this estrus-phase reduction, which was also observed in rats treated with ICI, is related to some impact of liquid infusions into LH and/or minor damage caused by the repeated infusion procedures, which potentially can disrupt LH activity enough to make the rats less sensitive to taste palatability when estradiol circulation is at its lowest. Regardless of its origin, this unexpected confounding effect of DMSO infusion did not obscure the magnification of palatability observed during metestrus, which shifted to diestrus following blockade of LH estrogen receptors. Thus, the infusion procedure appears to exert a quantitative, rather than qualitative, influence on palatability magnification. Future experiments employing chemogenetic inhibition instead of intracranial infusions will disentangle the specific contributions of infusion-related effects during estrus.

Regardless of the remaining questions, these results have implications for our understanding of the psychological effects of hormone changes across the human menstrual cycle, in that they suggest that the impact of estrous phase should not be considered in isolation for a single taste or even for a particular class of tastes (e.g., palatable, nutritive, or aversive). In natural environments, taste stimuli rarely occur alone but are typically experienced in combination with other tastes and often with odors. As shown here and in previous work involving contrast effects (Flaherty and Rowan, 1986; Flaherty et al., 1995; Ballintyn et al., 2023), alterations in consumption of one taste is likely to be a facet of changed processing of all tastes. When designing interventions to alleviate estrous-related alterations in feeding behavior, the combination of tastes may be a critical mediating factor determining treatment efficacy. A parametric approach will likely be necessary to optimize such treatment designs.

In summary, our results support the hypothesis that a modulation of taste palatability mediates the impact of estrous phase on food consumption. Thus, palatability depends not only on taste valence but also on the surrounding hormonal environment, in a manner best detected in within-subject behavioral designs. These findings also offer a framework for understanding cycle-linked changes in food craving and altered eating patterns in women experiencing disrupted ovarian hormone signaling, such as those taking oral contraceptives (Bancroft and Rennie, 1993; Freeman et al., 2001; Marr et al., 2011) or those with menstrual irregularities arising from conditions such as excessive exercise or disordered eating (Coppi et al., 2021).

## Synthesis

Reviewing Editor: Arianna Maffei, Stony Brook University Decisions are customarily a result of the Reviewing Editor and the peer reviewers coming together and discussing their recommendations until a consensus is reached. When revisions are invited, a fact-based synthesis statement explaining their decision and outlining what is needed to prepare a revision will be listed below. The following reviewer(s) agreed to reveal their identity: Anan Moran, Hillary Schiff.

The reviewers agree that this study addresses an important question in the field and that the results are potentially impactful. However, they have identified a number of concerns regarding the experimental design and analysis.

The sample size for several experiments is low and insufficient to achieve statistical power for the data analysis. Some labels and statistics are missing from the figures (see details in specific comments below). Confounding effects of DMSO are not controlled for, raising some concerns about the interpretation of the data. There is some concern about order effect in the brief access test, with a possible influence of the stage of the estrous cycle at the beginning of training. This is especially a concern regarding the data presented in Figure 6.

Finally, the reviewers identified several points in the experimental design and analyses that require clarifications.

Reviewer #1

The study provides valuable insights into the relationship between the estrous cycle, taste palatability, and hypothalamic estradiol signaling. The experimental design is robust, and the findings are significant for understanding hormonal influences on food consumption. However, there are several areas where the manuscript could be improved or clarified.

Strengths:

Novelty: The study addresses a critical gap in understanding how hormonal fluctuations impact central taste palatability processing, rather than just consumption behavior.

Experimental Design: The use of the Brief Access Test (BAT) to isolate palatability effects from post-ingestive factors is innovative and well-justified. The comparison to long-term (15 minutes) consumption from a single bottle is convincing.

Comprehensive Analysis: The study employs multiple statistical methods (ANOVA, GLMM, bootstrapping, chi-squared tests) to validate findings, ensuring rigor.

________________________________________

Weak Points and Suggestions for Improvement:

1. Lack of Clarity in Estradiol's Role

Issue: The study concludes that estradiol signaling in the LH modulates palatability, but the delayed effect observed with ICI infusions (shifting magnification to diestrus) is not fully explained. The authors indeed give some references to 24-48-hour delays that are known in estradiol cycles; a more mechanistic explanation, or at least a suggestion for such a mechanism, is essential. In the results (line 530), they claim that the effect on the metestrus phase originates from impact 2 days earlier in the diestrus phase. This assumption is highly speculative. To test this, the authors should have done a phase-specific inhibition.

Suggestion: Leave the assumption of the day of impact to the discussion. Provide a more detailed discussion of potential mechanisms behind the delayed effect. Consider exploring interactions with other hormones (e.g., progesterone, luteinizing hormone) or compensatory neural circuits.

2. Confounding Effects of DMSO

Issue: DMSO infusions appear to reduce palatability differences during estrus. The authors address this issue in the discussion, but nevertheless, this casts doubts on the infusion procedure as a blocking-only effect.

3. Statistical Power and Sample Size

Issue: The number of rats per estrous phase is somewhat low (e.g., only 4 rats in metestrus for Experiment 1), which may affect confidence in the results. It is even more problematic since the reported main effect was in this phase. This issue, however, is relaxed to some extent given the similarity to the surgereized rats of experiment 2 with DMSO, but again, there are also differences.

Suggestion: Increase sample sizes for underrepresented phases to ensure balanced data and improve the robustness of statistical analyses, or raise this issue in the discussion.

4. Limited References to Human Studies and Implications

Issue: While the study briefly mentions human relevance (e.g., menstrual cycle, menopause), the connection to human physiology and behavior is underdeveloped.

Suggestion: Expand the discussion on how these findings could inform human studies, particularly in the context of eating disorders, hormonal therapies, or menopause-related changes.

________________________________________

Minor Points:

1. Figures and Data Presentation

Issue: Some figures lack units on their axes, making interpretation difficult. Also, it is common now to add dots in bar graphs that represent single animal's values, thus giving more information about the source of the error bars presented.

Suggestion: Self-explanatory.

2. Line 90: "ER" not defined earlier.

3. Line 155: "gently"

4. Fig 2Bi and 2Bii: It is better to show the trends for each rat, not the mean over the group. The way it is analyzed now only shows that, on average, the palatabilities follow the same trend. The authors want to conclude, however, that this trend is true for most, if not all, rats.

5. Line 375: "conclude that these data provide a valid measure of palatabilities across an entire taste battery in single sessions, clearing the way for its use to compare palatability processing in different phases of the estrous cycle." However, these tests were made in different estrous phases, which you show have an impact on palatability. Resolve this issue.

6. Line 406: In Figure 3, you first show the lick cluster size for the palatable tastes in relation to the phases, then do a correlation between the pattern of all tastes and show it in Figure 3C, without showing the pattern of cluster size for the aversive tastes. In line 406, you come to the conclusion that "the palatability of all five tastes depends upon estrous phase, ...". I suggest you present the pattern for the aversive taste before such a claim.

________________________________________

Conclusion:

The manuscript is a strong contribution to the field, but addressing the above weaknesses would improve its clarity, rigor, and impact. Specifically, more detailed explanations of estradiol's delayed effects, estrus-phase suppression, and DMSO confounds are needed. Expanding the discussion on human relevance and improving data presentation would also enhance the manuscript's accessibility and significance.

Reviewer #2

The authors describe an important set of studies examining the role of the estrous cycle in taste palatability. The studies provide information related to previously documented changes in food consumption, which may be based on changes in taste perception and palatability, during the estrous cycle. Understanding differences in taste during the estrous cycle is an important and impactful area to study as researchers incorporate sex as a biological variable into chemosensory studies. The data presented in this manuscript contribute information to do so in a biologically sound way.

The authors first correlated licking behavior on a brief access and long access test, finding strong correlations in the size of lick clusters for three tastants on each test. This finding motivated them to analyze lick cluster size during brief access licking rather than total licking during the brief access test. The authors then examine the interaction of estrous cycle and palatability. They showed that for multiple palatable tastes (saccharin, sucrose, or salt), lick cluster size is largest during the metestrus phase compared to diestrus. Further analyses revealed that the difference in lick cluster size between palatable and aversive tastes was greatest during metestrus, supporting the notion that the range of palatability is larger during metestrus than other phases. Antagonizing estradiol in the lateral hypothalamus changed the phase in which the magnification of palatability range was detected. Although the mechanism underlying this shift in palatability is not elucidated, several reasonable possibilities are suggested in the discussion.

While the work is sufficiently impactful and much of the data are sound, I have major and minor concerns with the experimental design, presentation of results, and interpretation of data.

Major concerns:

1. My biggest concern with the experimental design is potential order effects. The order in which novel taste are presented in the Davis Rig plays a big role in the initial and subsequent responses to those tastants. Did the authors control during which phase the first BAT presentations occurred? If so, it is not clearly stated. I do not believe it is possible to have separate groups starting the training in each of 4 phases with the low n's presented here. I would suggest the authors run further studies with separate groups of rats starting in each phase. If they feel confident that the effects occur during metestrus based on the studies presented here, perhaps they could run only 2 additional groups with one beginning in proestrus (the tastes will no longer be novel during metestrus) and the other beginning in metestrus (the tastes will be novel during metestrus). If order effects are not controlled, they might completely account for the "phase-specific" change in palatability. This confound is particularly concerning given the shift in palatability difference found in Figure 6 following estradiol antagonist treatment. I understand this is a big ask because the phase is only known after the fact. The authors at least need to clarify what phase each rat was in during the initial exposure to tastes.

2. The data in Figure 3A are very nice-they clearly show the overall effect. But I am still not completely sure what is presented here-is this average cluster size for all clusters in a 10-s trial or only the size of the 1st cluster? For palatable tastes in thirsty animals, these are usually the same or very similar. Why not show us the total lick data from the brief access test for all the tastes tested rather than only as cluster size? These could be presented as raw licks (which might be quite similar to what is currently in 3A) or relative to water (normalized or water-subtracted). Presenting the palatable and aversive tastes together would be informative regarding the animals' behavior and interaction with each taste in a single session. I noted that the authors claim that "more sensitive measures are available" in the brief access data in the methods section, but the data would be more interpretable if total licks were also presented. Finally, the data presented in 3A would be bolstered by overlaying individual data points for each rat in addition to the mean{plus minus}SEM.

3. I am not sure I understand what is presented in Figure 3C. From reading the manuscript, I believe that this is showing that lick cluster size for each of 5 tastants presented during the BAT are positively correlated across days and across different phases of the estrous cycle. The panel suggests that each tastant is highly correlated with itself. But the rest of the figure, indeed the title of the figure, suggests that "Estrous phase impacts palatability". If palatability is impacted by estrous phase, why are the correlations not changing across phase?

4. Figure 2B shows an example individual rat. Then Figure 2C shows the population data but only as correlations. Can we please see the population data as presented in 2B? Are each taste correlated? If data are sufficient, I would suggest showing the correlations for each of the 3 tastes. Also in Figure 2C, it is not clear to me what "mbout_size" is or how it was calculated. The left bar is labeled "licks" but I believe the data are cluster size. If this is indeed the case, please re-label the graph.

5. Figure 4. Perhaps I misunderstand the formula used to normalize the cluster size. The methods state that it takes the size of a lick cluster at a given trial for a given taste and divides that by the mean cluster size for that taste for all trials across all phases. If this is the case, the average licks should center around 1 (actually, 100 because the formula states that this value is multiplied by 100). Why is this not the case for aversive tastes? The methods section states it can produce asymmetry, but it is not clear to me why. I also do not understand the analysis that led to the data presented in figure 4B. Please explain how the deviation values were determined. What are the open dots on this graph?

6. The water consumption data in Figure 7 would be more convincing if presented as water consumed normalized to all liquid consumed. However, I was not sure whether these values came from licking during the BAT or if there was a homecage manipulation. If these are from BAT, why is it presented as grams rather than number of licks? This is an important control-in addition to addressing volume consumed, it also speaks to my concern in point #2 that it would be good for readers to be able to observe the rats' licking to palatable and aversive tastes.

7. The authors conclude that taste palatability is altered by LH estradiol in a fashion that is delayed by a few days after estradiol peak, resulting in the magnification of palatability range during metestrus. Is the converse possible-that estradiol actually exerts its effects on every other phase, while metestrus is the only phase unaffected by estradiol? If there are literature examining taste palatability in ovariectomized rodents, can it be determined whether their behavior is more akin to metestrus or not-metestrus phases? I understand that replicating the behavior in ovariectomized females may be beyond the scope of the current project, but perhaps the authors could complete their analyses in unoperated male rats to determine whether non-cycling animals display the larger range of palatability like in metestrus or the more restricted range as in the other phases. This information could help clarify the finding of the delayed effect of blocking estradiol.

Minor Concerns:

1. The authors state that each rat received 10 intra-LH infusions of 1.0µl volume. This is both a large volume and a large number of intracranial infusions. I am not sure I have ever seen this many infusions undertaken in a single animal. Can the authors provide more sample images of cannulated rats' brains to confirm the lack of damage?

2. In Figure 3B, what does the significance asterisk represent? Were statistics ran for each tastant (sucrose, saccharin, NaCl) and compared from metestrus vs diestrus or was the comparison an average of all 3 palatable tastes? The statistical analyses run here are not clear, but it is important because the text reads "lick clusters produced to both sucrose and NaCl were largest during metestrus and lowest during diestrus" (lines 387-388) which leads me to believe that each group was significantly different across these 2 estrus phases. Please clarify and provide statistics.

3. Since the concentrations of tastants used for experiments presented in Figure 2 are different from those presented in Figure 3 (and the rest of the studies), please note the concentrations in the figure and/or figure legend.

4. The authors state that they chose to use 500 msec as the ILI cut-off for a lick cluster because it is "more than twice of the average ILI" (line 346). The average ILI is stated to be "approximately 120 ms". It is technically correct that 500 ms is more than 240 ms (2*120), but then why not choose 250ms as the cut-off? Using 500 ms is indeed a field standard, and so the justification seems unnecessary.

5. Figure legend for 2B states that it shows mean{plus minus}SEM but in the text, it is referred to as confidence intervals (line 354). Which is it?

6. Line 388, why is the parenthetical statement "(although the differences between diestrus and proestrus were small)" there? The previous statements only show significant differences between metestrus and diestrus. The parenthetical is confusing at best. There appears to be no difference at all between diestrus and proestrus. Please remove this.

7. Please combine figures 5 and 6 into a single figure.

8. Lines 459-460 state that a X2 test will be run "(see methods)" but I couldn't find a description of this test in the methods.

9. Some phrasing should be edited. Here are a few examples: "which suggests extremely high similarity" (lines 366-367); I think "suggests high similarity" is sufficient. Line 597: "it is highly likely that"; again, just "it is likely that" would be sufficient. In line 519, "it was of little statistical significance". The authors shouldn't decide what is or is not significant-that is what statistical tests are for. I think just saying that they do not have a conclusive explanation is sufficient.

## Author Response

We are grateful to the reviewers for their thoughtful and constructive criticisms (and to the editors for considering this revision). We have responded to each of the concerns, a move that required additional data collection; we hope that we can be forgiven for the resultant long turnaround of the manuscript-it was a necessary part of taking the reviews very seriously, and the manuscript is much stronger for it. Below, please find our detailed responses, with specific changes made highlighted with underlining and accompanied by references to where they can be found in the manuscript.

Reviewer #1 ("R1").

R1: "The study concludes that estradiol signaling in the LH modulates palatability...but the delayed effect observed with ICI infusions (shifting magnification to diestrus) is not fully explained; ...a more mechanistic explanation, or at least a suggestion for such a mechanism, is essential. The claim that the effect on the metestrus phase originates from impact 2 days earlier in the diestrus phase...is highly speculative." Response: R1 is quite correct; we can offer only speculative explanations for the complicated estrogen receptor inhibition results. Alas, the kind of phase-specific inhibition experiment that would permit tests of more mechanistic explanations would be quite complicated, lengthy, and expensive of animals (a full subject group for each phase, plus a lot of extra subjects that prove unusable because estrous phase is determined after the fact). Given that we have added new subjects to Experiment 1 (see below), which made the turnaround time on this revision very long, here we have (gratefully) taken R1's advice for an alternative solution by greatly expanding our discussion of potential mechanisms (including a deeper dive into possible interactions with other hormones and circuits; lines 692-712).

R1: "DMSO infusions appear to reduce palatability differences during estrus. The authors address this issue in the discussion, but nevertheless, this casts doubts on the infusion procedure as a blocking-only effect." Response: We have added further consideration of this issue to the above-mentioned Discussion (lines 719-725).

R1: "The number of rats per estrous phase is somewhat low (e.g., only 4 rats in metestrus for Experiment 1), which may affect confidence in the results. It is even more problematic since the reported main effect was in this phase. This issue, however, is relaxed to some extent given the similarity to the surgerized rats of experiment 2 with DMSO, but again, there are also differences." Response: When you're right, you're right, and R1 is right. For the resubmission, we have run an additional squad of rats for Experiment 1. These additional subjects increase the robustness of our Results, shoring up our confidence in both our interpretation of these data and of our comparison to the DMSO data, and also enable us to perform a novel analysis of session order (see below).

R1: "While the study briefly mentions human relevance (e.g., menstrual cycle, menopause), the connection to human physiology and behavior is underdeveloped." Response: We have taken R1's advice and added prose at two points in the Discussion regarding human-subjects research (lines 604-613, lines 726-736)-both the implications of our work for understanding human condition, and additional research on human subjects that supports our work.

R1 Less Central Points:

1. "Some figures lack units on their axes, making interpretation difficult. Also, it is common now to add dots in bar graphs that represent single animal's values, thus giving more information about the source of the error bars presented." Response: The requested changes have been made.

2. Line 90: "'ER' not defined earlier." Response: ER is now defined with first use (line 86).

3. Line 155: "gently" Response: Fixed.

4. "Fig 2Bi and 2Bii: It is better to show the trends for each rat, not the mean over the group. The way it is analyzed now only shows that, on average, the palatabilities follow the same trend. The authors want to conclude, however, that this trend is true for most, if not all, rats." Response: We have added the requested panel (now Figure 2C). We hasten to note, however, that the central hypothesis being tested with the data presented in Figure 2 is largely independent of what is shown in the new panel (i.e., that different rats show similar preferences); it would be possible that rats could show strong individual differences in what tastes they prefer (which would result in a lot of variability in Figure 2C), but that those individual differences would be reflected in both brief and 15-min tasting trials-resulting in the same significant correlations shown in Figure 2D, and validating our phase-to-phase analysis of preferences in the BAT rig.

5. "'...conclude that these data provide a valid measure of palatabilities across an entire taste battery in single sessions, clearing the way for its use to compare palatability processing in different phases of the estrous cycle.' However, these tests were made in different estrous phases, which you show have an impact on palatability. Resolve this issue." Response: We are happy to do so. The hypothesis being tested with the data presented in Fig. 2-that preference patterns is observed in brief-access trials will be observed in ad lib drinking trials-is affirmed by the correlation analysis (now Figure 2D). The cycle-related effects that we go on to study in the rest of the manuscript are changes in magnification of preferences, rather than wholesale changes in what is preferred) that coexist quite comfortably with the overall high correlations between sessions-we would see high correlations as long as there aren't wholesale changes in which tastes the rats prefer from phase to phase. It's also worth noting that 3 of the phases in the 4-day cycle are quite similar to one another with regard to preferences (see Fig. 4), which further explains the robustness of the Fig. 2D results.

6. "In Figure 3, you first show the lick cluster size for the palatable tastes in relation to the phases, then do a correlation between the pattern of all tastes and show it in Figure 3C, without showing the pattern of cluster size for the aversive tastes. In line 406, you come to the conclusion that 'the palatability of all five tastes depends upon estrous phase, ...'. I suggest you present the pattern for the aversive taste before such a claim." Response: The aversive taste pattern is now presented as part of Figure 3.

Reviewer #2 ("R2").

R2: "My biggest concern with the experimental design is potential order effects...Did the authors control during which phase the first BAT presentations occurred? If so, it is not clearly stated...If order effects are not controlled, they might completely account for the "phase-specific" change in palatability. This confound is particularly concerning given the shift in palatability difference found in Figure 6 following estradiol antagonist treatment. I understand this is a big ask because the phase is only known after the fact." Response: A "big ask," perhaps, but also an entirely reasonable concern (one that we're embarrassed not to have thought of), particularly given that Day 1 data was not uniformly spread between phases. And the Experiment 1 dataset was, in the original submission, a bit small for confident interpretation of such analyses. And so we decided, quite simply, to collect additional data: obviously, and as noted by R2, it wasn't possible to build control for order effects into the design (because phase is only known after the fact), but with that additional data we were able to do a robust re-analysis of our data in terms of order effects; with this analysis (which is now Figure 5; lines 485-504), we can more confidently report that the basic phenomenology-the metestrus-specific magnification of palatability differences-does not reflect variables (i.e., attenuation of neophobia) having to do with order of test.

R2: "In Figure 3A...is this average cluster size for all clusters in a 10-s trial or only the size of the 1st cluster? Why not show us the total lick data from the brief access test for all the tastes tested rather than only as cluster size?...Finally, the data presented in 3A would be bolstered by overlaying individual data points for each rat in addition to the mean {plus minus} SEM." Response: Figure 3A shows average lick cluster size, which we used because it provides more sturdy, robust estimates of palatability than total licking (as we note in our Methods section as well as demonstrated in Figure 2), particularly for small datasets. It is the measure used in Figures 4 and 6 as well, and its use here adds to the consistency of the figure set. But in appreciation of R2's concern, we have added an inset to Figure 3A (and B) that shows that lick cluster size simply provides a more robust version of what is also visible in total per-trial lick measurement (lines 397-403).

R2: "I am not sure I understand what is presented in Figure 3C. From reading the manuscript, I believe that this is showing that lick cluster size for each of 5 tastants presented during the BAT are positively correlated across days and across different phases of the estrous cycle. The panel suggests that each tastant is highly correlated with itself. But the rest of the figure, indeed the title of the figure, suggests that 'Estrous phase impacts palatability'. If palatability is impacted by estrous phase, why are the correlations not changing across phase?" Response: We apologize for having done a poor job of explaining and presenting this panel. It has been replaced by a pair of bar graphs (Figure 3C1 and 2) that more clearly make the point (lines 403-412) that phase-to-phase changes in preferences for tastes of similar palatability are similar despite vast inter-stimulus differences (thereby justifying the decision to proceed with analyses that collapse the data into "palatable" and "aversive" tastes.

R2: "Figure 2B shows an example individual rat. Then Figure 2C shows the population data but only as correlations. Can we please see the population data as presented in 2B? ... I would suggest showing the correlations for each of the 3 tastes. Also in Figure 2C...please re-label the graph [to be more clear]." Response: We have added the requested panel showing across-rat average licking (i.e., the group data in the form of Figure 2B)-this analysis is now Figure 2C. We have retained Figure 2C (which is now Figure 2D) as well (because it is the more direct test of our hypothesis that individual rats' taste preference patterns are similar in paradigms with 10-sec and 15-min availability), but have changed the Figure 2D axis labels to be more clearly informative. Together, these panels demonstrate that preference patterns (which are ultimately what we go on to examine with the rest of the manuscript) are in fact the same in the two paradigms. We have not gone on to perform additional single-taste analyses-testing. Instead, we add data points from each rats (as a scatter plot) to show that the brief- and long-trial session correlations are consistently high across rats. But if R2 feels that such analyses would add important information, we will happily attempt them.

R2: "Perhaps I misunderstand the formula used to normalize the cluster size. The methods state that it takes the size of a lick cluster at a given trial for a given taste and divides that by the mean cluster size for that taste for all trials across all phases. If this is the case, the average licks should center around 1 (actually, 100 because the formula states that this value is multiplied by 100). Why is this not the case for aversive tastes? The methods section states it can produce asymmetry, but it is not clear to me why. I also do not understand the analysis that led to the data presented in figure 4B. Please explain how the deviation values were determined. What are the open dots on this graph?" Response: Thank you for pointing out the misrepresentation of the formula. We have now corrected it by removing the "×100" term (line 220). The reviewer is absolutely right that, based on the normalization formula, the overall average lick cluster size should be one under ideal conditions-i.e., when data from each rat are available for all phases. However, in practice, we did not have exactly the same number of rats in each phase across all experiments, as it was not always possible to definitively identify the estrous phase. As a result, normalization performed within each rat can yield average scores below 1.

We have revised the text to clarify 1) The step-by-step procedure (lines 451-455) used to compute deviation scores for each rat, and the use of one-sample t-tests to assess whether those deviations significantly differ from the population mean (which is 1 in this case) and 2) Why the normalization method may yield asymmetrical values for enhancement (values above the phase average) and suppression (values below the phase average) (lines 227-230).

R2: "The water consumption data in Figure 7 would be more convincing if presented as water consumed normalized to all liquid consumed. However, I was not sure whether these values came from licking during the BAT or if there was a homecage manipulation. If these are from BAT, why is it presented as grams rather than number of licks? This is an important control-in addition to addressing volume consumed, it also speaks to my concern in point #2 that it would be good for readers to be able to observe the rats' licking to palatable and aversive tastes." Response: The water consumption data were collected as part of the BAT, where water was included as one of the stimuli in the taste battery. These data have now been converted to ratios relative to other fluid consumption (lines 590-597), as requested. We chose to present water consumption in grams rather than in number of licks because the amount of fluid consumed per lick is not constant-it varies across conditions, particularly when comparing tastes with different palatability. Specifically, rats tend to consume more fluid per lick for palatable tastes and less for aversive ones. As such, using grams provides a more accurate measure of actual intake. This allowed us to better assess whether general fluid motivation (as indexed by water consumption) fluctuates with the estrous cycle.

R2: "Is [it] possible that estradiol actually exerts its effects on every other phase, while metestrus is the only phase unaffected? ... I understand that replicating the behavior in ovariectomized females may be beyond the scope of the current project, but perhaps the authors could complete their analyses in unoperated male rats to determine whether non-cycling animals display the larger range of palatability like in metestrus or the more restricted range as in the other phases.

Response: This is a very intriguing question. We have indeed run a small sample (N = 4) of male rats that received multiple consecutive BAT rig tests, but analysis of these data would be inconclusive even if they were substantial enough to present on their own (see below). But we can speculate that several pieces of evidence suggest that the answer must be "both" (sex hormones enhance metestrus palatability processing AND suppress palatability processing in other phases), and we do so at some length in the Discussion (line 621-630).

R2 Minor Concerns:

1. "The authors state that each rat received 10 intra-LH infusions of 1.0µl volume. This is both a large volume and a large number of intracranial infusions. I am not sure I have ever seen this many infusions undertaken in a single animal. Can the authors provide more sample images of cannulated rats' brains to confirm the lack of damage?" Response: To minimize potential damage to LH from the infusions, we implanted the guide cannulae such that the injectors targeted a position just lateral to the nucleus rather than within the nucleus itself (see histology figure); pilot testing revealed that it is very difficult to avoid causing injection-related LH damage when injection cannulae are within LH itself. Given that the infusate therefore needed to diffuse a short distance to LH, we increased the dosage somewhat. Our results confirm the success of this procedure: had diffusion not occurred, we would have seen no effect of ICI injection; had tissue damage been a problem, meanwhile, we would have seen little or no difference between the DMSO and ICI conditions. We now make these points in the manuscript.

2. "In Figure 3B, what does the significance asterisk represent? Were statistics ran for each tastant (sucrose, saccharin, NaCl) and compared from metestrus vs diestrus or was the comparison an average of all 3 palatable tastes? The statistical analyses run here are not clear, but it is important because the text reads 'lick clusters produced to both sucrose and NaCl were largest during metestrus and lowest during diestrus' (lines 387-388) which leads me to believe that each group was significantly different across these 2 estrus phases. Please clarify and provide statistics." Response: We have removed this panel and generally clarified the statistics in use elsewhere.

3. "Since the concentrations of tastants used for experiments presented in Figure 2 are different from those presented in Figure 3 (and the rest of the studies), please note the concentrations in the figure and/or figure legend." Response: These have been added to the Figure 2 caption (lines 1022-1023).

4. "The authors state that they chose to use 500 msec as the ILI cut-off for a lick cluster because it is 'more than twice of the average ILI' (line 346). The average ILI is stated to be "approximately 120 ms". It is technically correct that 500 ms is more than 240 ms (2*120), but then why not choose 250ms as the cut-off? Using 500 ms is indeed a field standard, and so the justification seems unnecessary.

Response: Fixed, 5. "Figure legend for 2B states that it shows mean{plus minus}SEM but in the text, it is referred to as confidence intervals (line 354). Which is it?" Response: We have fixed the terminology confusion.

6. "Line 388, why is the parenthetical statement '(although the differences between diestrus and proestrus were small)' there? The previous statements only show significant differences between metestrus and diestrus. The parenthetical is confusing at best. There appears to be no difference at all between diestrus and proestrus. Please remove this." Response: Done! 7. "Please combine figures 5 and 6 into a single figure." Response: Done.

8. "Lines 459-460 state that a X2 test will be run "(see methods)" but I couldn't find a description of this test in the methods." Response: Text changed (lines 289-291).

9. "Some phrasing should be edited. Here are a few examples: 'which suggests extremely high similarity' (lines 366-367); I think 'suggests high similarity' is sufficient. Line 597: 'it is highly likely that'; again, just 'it is likely that' would be sufficient. In line 519, 'it was of little statistical significance'. The authors shouldn't decide what is or is not significant-that is what statistical tests are for. I think just saying that they do not have a conclusive explanation is sufficient." Response: All changes made, with apologies for our overexuberance.
